# AvrBsT Acetylates *Arabidopsis* ACIP1, a Protein that Associates with Microtubules and Is Required for Immunity

**DOI:** 10.1371/journal.ppat.1003952

**Published:** 2014-02-20

**Authors:** Mi Sun Cheong, Angela Kirik, Jung-Gun Kim, Kenneth Frame, Viktor Kirik, Mary Beth Mudgett

**Affiliations:** 1 Department of Biology, Stanford University, Stanford, California, United States of America; 2 School of Biological Sciences, Illinois State University, Normal, Illinois, United States of America; The University of North Carolina at Chapel Hill, United States of America

## Abstract

Bacterial pathogens of plant and animals share a homologous group of virulence factors, referred to as the YopJ effector family, which are translocated by the type III secretion (T3S) system into host cells during infection. Recent work indicates that some of these effectors encode acetyltransferases that suppress host immunity. The YopJ-like protein AvrBsT is known to activate effector-triggered immunity (ETI) in *Arabidopsis thaliana* Pi-0 plants; however, the nature of its enzymatic activity and host target(s) has remained elusive. Here we report that AvrBsT possesses acetyltransferase activity and acetylates ACIP1 (for *ACETYLATED INTERACTING PROTEIN1*), an unknown protein from *Arabidopsis*. Genetic studies revealed that *Arabidopsis* ACIP family members are required for both pathogen-associated molecular pattern (PAMP)-triggered immunity and AvrBsT-triggered ETI during *Pseudomonas syringae* pathovar *tomato* DC3000 (Pst DC3000) infection. Microscopy studies revealed that ACIP1 is associated with punctae on the cell cortex and some of these punctae co-localize with microtubules. These structures were dramatically altered during infection. Pst DC3000 or Pst DC3000 AvrRpt2 infection triggered the formation of numerous, small ACIP1 punctae and rods. By contrast, Pst DC3000 AvrBsT infection primarily triggered the formation of large GFP-ACIP1 aggregates, in an acetyltransferase-dependent manner. Our data reveal that members of the ACIP family are new components of the defense machinery required for anti-bacterial immunity. They also suggest that AvrBsT-dependent acetylation *in planta* alters ACIP1's defense function, which is linked to the activation of ETI.

## Author Summary

How host disease resistance pathways are activated in response to pathogens remains a fundamental question in host-pathogen interactions. In this work, we used the *Pseudomonas-Arabidopsis* pathosystem to study how the AvrBsT effector activates plant immune signaling. AvrBsT belongs to the YopJ effector family, a group of virulence proteins shared by bacterial pathogens of plants and animals. Bacteria inject these effectors into plant or animal host cells to promote pathogenesis. Recent biochemical studies show that several members of the YopJ family encode acetyltransferases that acetylate host proteins to suppress immune signaling. How the immune system specifically recognizes this family of effectors and/or monitors host acetylation is poorly understood. In this work, we provide biochemical evidence that AvrBsT is an acetyltransferase. We also report the identification and characterization of ACIP1, an *Arabidopsis* protein of unknown function that is an AvrBsT substrate. We provide evidence that ACIP1 is required for plant immunity and its association with microtubules changes during infection. Moreover, our work suggests that AvrBsT acetyltransferase *in planta* leads to dramatic changes in ACIP1 localization, which coincides with the activation of strong defense responses. This study highlights an important link between ACIP1 and the microtubule network during anti-bacterial immunity.

## Introduction

It is well established that bacterial pathogens utilize type III secretion (T3S) systems to translocate virulence factors (referred to as T3S effectors) into eukaryotic hosts to modulate immune signaling during infection [1]. The T3S effector proteome reflects the coevolution of specific host-pathogen interactions as well as microbe-microbe interactions within a given environment. Few T3S effector homologs are conserved among bacterial pathogens that colonize plant or animals hosts. One exception is the YopJ effector family, which is shared by a number of bacterial species in different genera (*e.g. Yersinia*, *Salmonella*, *Vibrio, Pseudomonas*, *Xanthomonas*, and *Sinorhizobium*) [2].

The YopJ effector family is named after the archetypal protein YopJ, first identified in *Yersinia pseudotuberculosis* [3]. These effectors belong to the C55 peptidase family because they share putative structural folds characteristic of cysteine proteases and contain the conserved catalytic triad – His, Glu and Cys [4]. Mutation of this catalytic triad destroyed effector-triggered phenotypes in host cells [5], providing the first clue that enzyme activity is critical for the virulence of the YopJ effector family. Biochemical studies revealed however that YopJ has potent acetyltransferase activity [6]. In subsequent work, several effectors from this family were shown to have acetyltransferase activity important for host-pathogen interactions, including VopA from *Vibrio parahemeolyticus* [7], AvrA from *Salmonella typhimurium* [8], PopP2 from *Ralstonia solanacearum* [9], and HopZ1a from *Pseudomonas syringae* [10]. These data indicate that a predominant virulence activity for the YopJ effector family is the post-translational acetylation of host proteins.

Resistance to YopJ-like effectors (*i.e.* AvrBsT, AvrRxv, AvrXv4, HopZ1a, and PopP2) has been reported in several plant hosts [11]–[13]; however, only two disease resistance (R) proteins have been characterized to date [14], [15]. *Arabidopsis* RRS1-R (for *RESISTANCE TO RALSTONIA SOLANACEARUM1*) is a Toll-IL-1-receptor-nucleotide binding site-leucine rich repeat-WRKY motif (TIR-NBS-LRR-WRKY)-type R protein that recognizes the PopP2 effector from *Ralstonia solanacearum* [14]. RRS1-R directly interacts with PopP2 in the plant nucleus [16]. *Arabidopsis* ZAR1 (for *HOPZ ACTIVATED RESISTANCE1*) is a coiled-coil (CC)-NBS-LRR-type disease R protein that recognizes the HopZ1a effector from *Pseudomonas syringae* and activates immune signaling that is distinct from most R protein pathways and independent of salicylic acid [15]. Neither RRS1-R nor ZAR1 were reported to be acetylated by the corresponding acetyltransferase [9], [10] suggesting that acetylation of other plant targets is required for recognition and/or initiation of defense signaling by these R proteins. Interestingly, a recent study revealed that HopZ1a acetylates the *Arabidopsis* ZED1 (for *HOPZ-ETI DEFICIENT1*), a pseudokinase that is required for ZAR1-mediated immunity [17]. ZED1 is proposed to act as a decoy in a ZAR1 defense complex.

Notably in mammals, YopJ acetylation suppresses innate immune signaling by exclusively targeting kinases in mitogen-activated protein kinase (MAPK) and/or NF–κB pathways. For example, YopJ catalyzes the O-acetylation of Ser or Thr residues in the activation loop of MAPKK6 [6], MEK2 [18], inhibitor of kappa B kinase [18], and MAP3K transforming growth factor β-activated kinase 1 (TAK1) [19]. Similarly in flies, AvrA inhibits c-Jun N-terminal kinase signaling by O-acetylation of the Thr residue in the activation loop of the MAPKK JNK-K [8].

In plants, a direct link between YopJ-like effector acetylation and suppression of disease resistance has not been made. HopZ1a was reported to acetylate tubulin *in vitro*, suggesting that the plant cytoskeleton may be disrupted during infection [10]. Consistent with this hypothesis, *P. syringae* pathovar *tomato* strain DC3000 (Pst DC3000) infection reduced microtubule density in a HopZ1a catalytic-dependent manner [10]. Interestingly, the mammalian tubulin acetyltransferase TAT1 acetylates Lys40 in α-tubulin (Nε-acetylation) [20], [21] and this modification is commonly found in less dynamic microtubules. The type of tubulin acetylation mediated by HopZ1a *in planta* has not yet been reported.

In previous work, we exploited the use of the *Pseudomonas-Arabidopsis* pathosystem to elucidate the biochemical function of the AvrBsT effector from *Xanthomonas euvesicatoria*. AvrBsT was engineered to be delivered into plant cells by Pst DC3000's T3S system [22] because *Arabidopsis* is not a host for *X. euvesicatoria*. Two *Arabidopsis* ecotypes were identified that differentially respond to Pst DC3000 AvrBsT infection. The Col-0 ecotype is susceptible to Pst DC3000 AvrBsT infection whereas the Pi-0 ecotype is resistant. Pi-0 resistance is due to a recessive, loss of function mutation in *SOBER1* (for *SUPPRESSOR OF AVRBST-ELICITED RESISTANCE1*). SOBER1 encodes a α/β-hydrolase that negatively regulates the accumulation of phosphatidic acid (PA) triggered by AvrBsT activity during bacterial infection [23]. High PA levels in Pst DC3000 AvrBsT-infected Pi-0 leaves correlate with ETI-like defense responses [22], [23]. These data suggest that AvrBsT interferes with lipid homeostasis during infection and that this interference induces strong immune responses in the absence of SOBER1 activity.

Given that PA is a multifunctional stress signal [24], we hypothesized that AvrBsT-triggered PA bursts may directly lead to the local activation of defense signaling. Moreover, we hypothesized that AvrBsT host targets may be linked to the generation or perception of lipid signals during AvrBsT-triggered immunity. To begin to test these hypotheses, we sought to identify AvrBsT interacting proteins from *Arabidopsis* and elucidate their function(s) in the Pi-0 *sober1-1* background [22]. Importantly, the availability of putative host substrates also enabled us to determine if AvrBsT possesses acetyltransferase activity, as reported for other effectors in the YopJ family [6], [9], [10].

Here we report that AvrBsT has acetyltransferase activity. We provide evidence that AvrBsT-dependent trans-acetylation activity is required for the activation of ETI in *Arabidopsis* Pi-0 leaves and that AvrBsT trans-acetylates *Arabidopsis* ACIP1 (for *ACETYLATED INTERACTING PROTEIN1*). ACIP1 is an unknown protein that localizes to punctae on the cell cortex and some of these punctae co-localize with cortical microtubules. We provide evidence that ACIP1 is a new component of the defense machinery required for anti-bacterial immunity. These data support the model that AvrBsT-dependent acetylation *in planta* alters ACIP1's defense function, which is linked to the activation of ETI.

## Results

### AvrBsT interacts with *Arabidopsis* ACIP1

**Figure 1:**
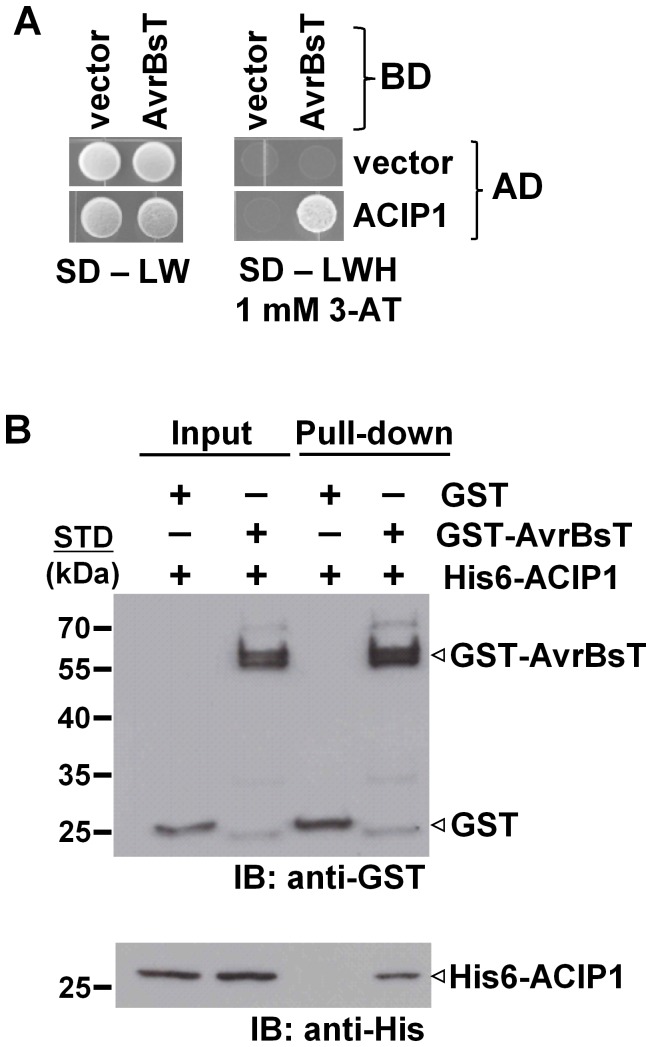
AvrBsT interacts with *Arabidopsis* ACIP1. (A) Yeast two-hybrid assay showing AvrBsT binding to *Arabidopsis* ACIP1. Yeast strain AH109 carrying pXDGATcy86 (vector) or pXDGATcy86(AvrBsT) were independently transformed with the pGADT7 (vector) or pGADT7 containing ACIP1. pXDGATcy86 contains the GAL4 DNA-binding domain (BD) and pGADT7 contains the GAL4 activation domain (AD). Strains were spotted on nonselective (SD – LW) and selective (SD –LWH +1 mM 3-AT) media and then incubated at 30°C for 3 d. (B) GST-AvrBsT affinity purification of His6-ACIP1 *in vitro*. GST or GST-AvrBsT was incubated with *E. coli* extracts containing His6-ACIP1. Proteins were purified by using glutathione sepharose and analyzed by immunoblot (IB) analysis using anti-GST and anti-His sera. Protein input is shown on left and pull-down on right. Expected protein MW: GST = 28 kDa; GST-AvrBsT = 65 kDa; and His6-ACIP1 = 28 kDa. +, protein expressed; −, vector control. STD, molecular weight standard. Similar phenotypes were observed in at least 3 independent experiments.

To identify potential AvrBsT-interacting proteins in *Arabidopsis*, we performed a yeast two-hybrid screen using the GAL4 DNA-binding domain (BD) fused to AvrBsT (*i.e.* BD-AvrBsT) and an *Arabidopsis* cDNA library fused to the GAL4 activation domain (AD). We screened ∼7 million primary yeast transformants and isolated 11 independent clones with a candidate cDNA encoded by At3g09980 (Figure 1A and S1A). Given that AvrBsT is predicted to encode an acetyltransferase, we named the At3g09980-encoded protein ACIP1, for putative acetylated-interacting protein 1 (Figure 1A).

ACIP1 is predicted to encode a protein with 178 amino acids and molecular weight of ∼20.6 kDa. ACIP1's only distinguishing feature is that it is predicted to be a small, α-helical protein [25] that contains the widely conserved domain of unknown function, DUF662 [26]. It was first identified as a tubulin-binding protein [27]. ACIP1 belongs to a small *Arabidopsis* protein family containing six ACIP-like isoforms (ACIP-L1 to ACIP-L6, Figure S1A). ACIP-L4 and its wheat ortholog TaSRG are required for salt tolerance [28], although their biochemical function(s) are not known. ACIP1 shares 79% identity and 87% similarity with ACIP-L1, the closest isoform. A tree for the *Arabidopsis* ACIP protein family is shown in Figure S1B. None of the ACIP-like isoforms were isolated in the primary AvrBsT interaction screen.

A candidate yeast interaction screen comparing AvrBsT binding to ACIP1 or the six ACIP-like isoforms revealed that AvrBsT strongly interacts with ACIP1 but only weakly interacts with ACIP-L1 on selection media containing 1 mM 3-AT (Figure S1C,D). In the presence of 5 mM 3-AT, AvrBsT only interacted with ACIP1 (data not shown). Taken together, these data suggest that AvrBsT preferentially binds to ACIP1 in yeast.

Next, we used GST pull-down assays to independently monitor the physical association of AvrBsT and ACIP1 *in vitro*. Recombinant GST and GST-AvrBsT were expressed in *E. coli* and then purified using glutathione sepharose. Purified GST-AvrBsT migrated as a doublet in protein gels, suggesting that proteolysis of the full-length polypeptide likely occurred during extraction and/or affinity purification. His-tagged ACIP1 was expressed in *E. coli* and soluble protein extracts were incubated with the GST or GST-AvrBsT in a standard GST pull-down assay. His6-ACIP1 was affinity purified by GST-AvrBsT but not GST (Figure 1B). These findings are in agreement with the yeast two-hybrid data and provide additional evidence that AvrBsT interacts with *Arabidopsis* ACIP1.

We attempted to verify AvrBsT-ACIP1 physical interaction *in planta*; however, the assays were not successful. Transient or inducible expression of AvrBsT in *Arabidopsis* Pi-0 leaves or *Nicotiana benthamiana* leaves results in localized cell death. It was difficult to obtain reproducible, conclusive binding data under these cellular conditions.

### AvrBsT has acetyltransferase activity

AvrBsT belongs to the YopJ family of T3S effector proteins, some of which have been shown to exhibit acetyltransferase activity [6], [9], [10]. To ascertain if AvrBsT acetylates ACIP1, we first sought to determine if AvrBsT possesses auto-acetylation activity *in vitro*. Recombinant wild-type GST-AvrBsT, GST (negative control) and GST-HopZ1a (positive control) [10] were over-expressed in *E. coli* and then purified using glutathione sepharose. Purified proteins were incubated with ^14^C-acetyl-coenzyme A (acetyl-CoA) ±100 nM inositol hexakisphosphate (IP_6_) for 30 minutes at room temperature and then separated by SDS-PAGE analysis followed by autoradiography. IP_6_ is a eukaryotic cofactor that stimulates the acetyltransferase activity of effectors in the YopJ family [9], [10], [29].

**Figure 2:**
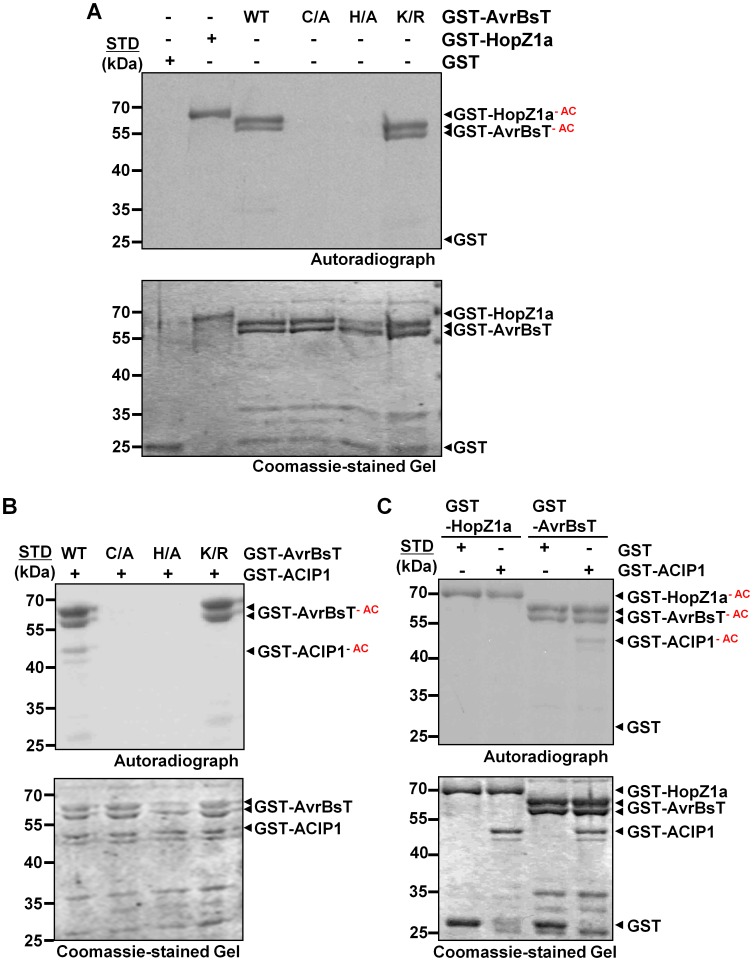
AvrBsT is an acetyltransferase that specifically acetylates ACIP1. (A) AvrBsT auto-acetylation activity *in vitro*. Acetylation reactions using GST and GST-AvrBsT (wild-type, C222A, H154A, and K282R) proteins. (B) AvrBsT trans-acetylates ACIP1 *in vitro*. Acetylation reactions using GST-ACIP1 or GST with GST-AvrBsT, GST-AvrBsT(C222A), GST-AvrBT(H154A) or GST-AvrBsT(K282R). (C) Substrate specificity of AvrBsT and HopZ1a. Acetylation reactions using GST-ACIP1 or GST with GST-HopZ1a or GST-AvrBsT. For acetylation reactions, proteins were incubated with 0.4 µCi ^14^C-acetyl CoA and 100 nM inositol hexakisphosphate (IP_6_) for 30 min at RT. Proteins were then separated by SDS-PAGE. Gels were stained with Coomassie and then analyzed by autoradiography. GST and GST-HopZ1a were used as negative and positive acetyltransferase enzyme controls, respectively. Acetylated proteins (GST-HopZ1a-^AC^, GST-AvrBsT-^AC^, and GST-ACIP1-^AC^) are labeled in the autoradiograph. STD, molecular weight standard in kDa. GST = 28 kDa; GST-HopZ1a = 70 kDa; GST-AvrBsT = 65 kDa; GST-ACIP1 = 50 kDa. Similar results were obtained in three independent experiments.

Auto-acetylation of GST-AvrBsT was detected in the presence of IP_6_ but not its absence (Figure 2A and S2). As expected, similar IP_6_-dependent activation and auto-acetylation of GST-HopZ1a was observed, and GST was not modified (Figure 2A and S2). Mutation of the conserved catalytic Cys residue (C222) or His residue (H154) to Ala inactivated AvrBsT-dependent acetyltransferase activity but did not affect protein expression levels (Figure 2A). By contrast, mutation of the conserved Lys residue (K282) (Figure S3A) to Arg, which has been shown to be an auto-acetylation site for some effectors in the YopJ family [9], [10], did not affect AvrBsT's acetylation state or protein accumulation (Figure 2A). The auto-acetylation activity of GST-AvrBsT(K282R) was comparable to that of wild-type GST-AvrBsT in reactions with varying concentrations of enzyme (Figure S3B). All GST-AvrBsT protein (wild type and mutant) analyzed migrated as a doublet and both of these species were auto-acetylated (Figure 2). Taken together, these data indicate that AvrBsT possesses auto-acetylation activity *in vitro* that is dependent on the conserved catalytic residues H154 and C222, but this activity is independent of K282.

### AvrBsT acetylates ACIP1 *in vitro*

Next, we tested if AvrBsT directly acetylates ACIP1 using similar reaction conditions to those described above. Wild-type GST-AvrBsT activity resulted in auto-acetylation of the enzyme and trans-acetylation of GST-ACIP1 (Figure 2B), whereas the catalytic core mutants GST-AvrBsT(C222A) or GST-AvrBsT(H154A) exhibited neither activities (Figure 2B). Although the GST-AvrBsT(K282R) mutant possessed auto-acetylation activity, trans-acetylation of ACIP1 was not detected under the same reaction conditions (Figure 2B). Importantly, mutation of C222 or K282 did not disrupt AvrBsT binding to ACIP1 *in vitro* (Figure S4).

To gain insight to the specificity of acetyltransferases in the YopJ effector family, we determined if HopZ1a could acetylate AvrBsT's substrate ACIP1. Conversely, we determined if AvrBsT could acetylate HopZ1a's substrate tubulin [10]. Incubation of GST-HopZ1a with GST-ACIP1 did not result in detectable acetylation of ACIP1 (Figure 2C). Moreover, neither HopZ1a nor other members of the HopZ family could physically associate with ACIP1 in targeted yeast two-hybrid screens (Figure S5A, B). Similarly, we could not detect AvrBsT-dependent acetylation of tubulin *in vitro* (Figure S5C) or direct physical interaction between AvrBsT and tubulin in yeast (Figure S5A,B). These data suggest that AvrBsT and HopZ1a possess distinct substrate specificity.

### AvrBsT(K282R) does not elicit resistance in Pi-0 leaves

**Figure 3:**
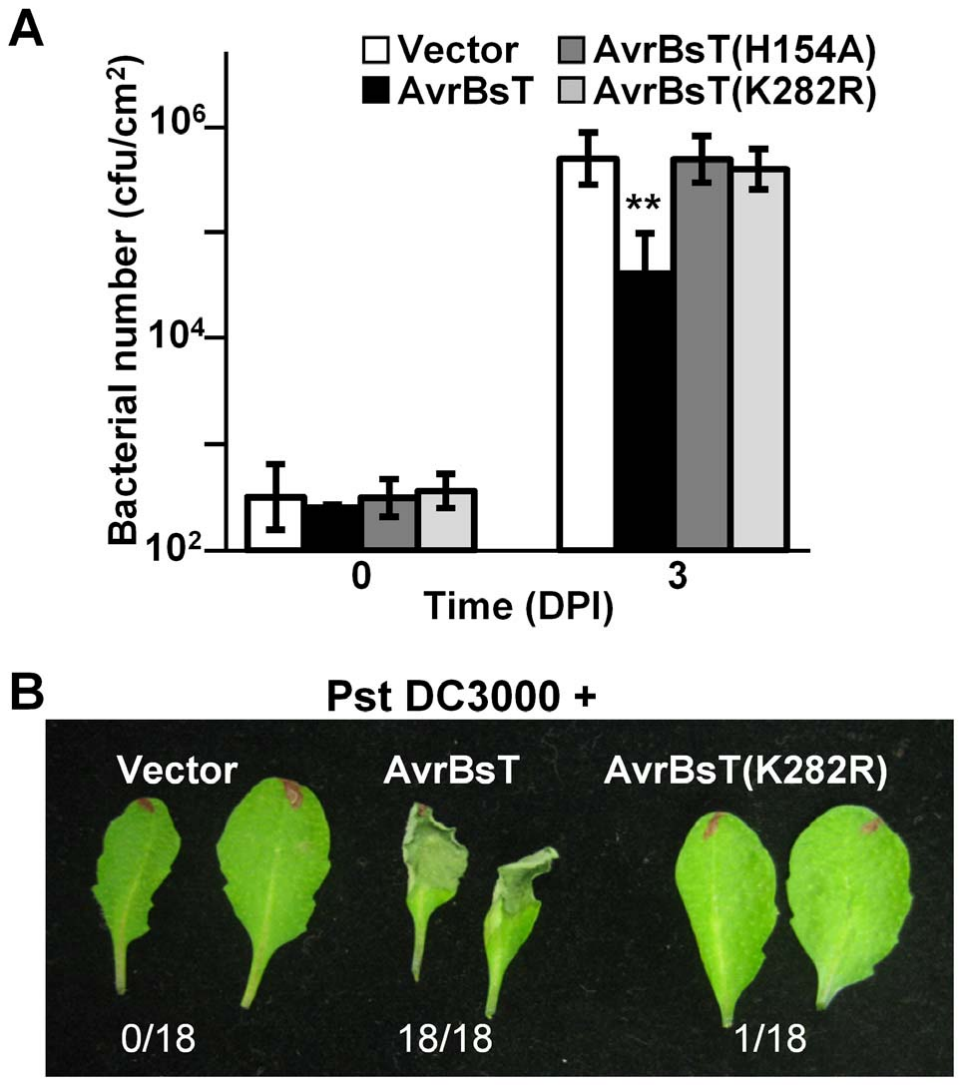
Mutation of K282 attenuates AvrBsT-triggered resistance. (A) Growth of Pst DC3000 in *Arabidopsis* Pi-0 leaves. Leaves were syringe infiltrated with a 1×10^5^ cells/mL suspension of bacteria: Pst DC3000 carrying vector (black bars), AvrBsT (white bars), AvrBsT(H154A) (dark grey bars) or AvrBsT(K282R) (light grey bars). Titers were assessed at 0 and 3 days post-inoculation. Data are mean cfu/cm^2^ ± SD (n = 6). Asterisks indicate statistically significant differences from Pi-0 (student's *t*-test, **p<0.01). Similar results were obtained in three independent experiments. (B) HR phenotypes in Pi-0 leaves. Leaves were infiltrated with a 3×10^8^ cells/mL suspension of Pst DC3000 carrying vector, AvrBsT or AvrBsT(K282R). Photograph was taken at 12 hours post-inoculation (HPI). Number of leaves exhibiting confluent HR at 10 HPI out of 18 inoculated leaves is shown at bottom.

We assessed the biological activity of the AvrBsT(K282R) mutant in *Arabidopsis* Pi-0 leaves, given that mutation of the analogous Lys residue in PopP2 and HopZ1a inhibits effector auto-acetylation activity and effector-dependent phenotypes *in planta* [9], [10]. Bacterial growth curve analyses showed that the K282R mutation attenuated the ability of AvrBsT to activate defense in Pi-0, similar to that observed for the H154A mutation in the catalytic core (Figure 3A). Furthermore, Pst DC3000 expressing AvrBsT(K282R) did not elicit HR in Pi-0 leaves (Figure 3B) despite stable protein expression (Figure S3C). These data indicate that the K282R mutation affects AvrBsT's trans-acetylation activity *in vitro* (Figure 2B) and its defense eliciting activity *in planta* (Figure 3). Moreover, these data indicate that the auto-acetylation activity of AvrBsT(K282R) is not sufficient to activate ETI in *Arabidopsis*.

### ACIP1 is a positive regulator of immunity

Given that nothing was known about ACIP1 function, we first sought to elucidate its potential role in immunity. Previously we showed that the Pi-0 ecotype of *Arabidopsis* is resistant to Pst DC3000 expressing AvrBsT, whereas the Col-0 ecotype is susceptible [22]. Interestingly, *ACIP1* mRNA abundance was significantly reduced at 3 and 6 HPI in Pi-0 (Figure S6A) and Col-0 (data not shown) leaves inoculated with a 2×10^8^ cells/mL suspension of Pst DC3000 or Pst DC3000 AvrBsT compared to leaves inoculated with mock solution of 1 mM MgCl_2_. By contrast, endogenous ACIP1 protein levels appeared to remain constant (Figure S6B). These data suggest that *ACIP1* may be transcriptionally or post-transcriptionally regulated during pathogen attack and potentially linked to PTI and/or ETI.

To explore this further, we first analyzed the growth of virulent Pst DC3000 in a homozygous Col-0 *acip1* null mutant (SALK_028810) to determine if ACIP1 is required to limit pathogen growth. Pst DC3000 grew equally well in wild-type Col-0 and *acip1* mutant leaves (data not shown). Similar results were observed when the *acip1* null allele was crossed into the Pi-0 background (data not shown). We speculated that the lack of a bacterial growth phenotype in the Col-0 *acip1* and Pi-0 *acip1* mutants may be due to genetic redundancy since *ACIP1* belongs to a small gene family in *Arabidopsis* (Figure S1A,B). Since the nucleotide sequences between *ACIP1* and *ACIP-like* genes are highly conserved (Figure S7A), we engineered RNAi lines to target multiple *ACIP* family members in attempt to uncover an immune phenotype linked to this gene family. Notably, we silenced the *ACIP* gene family in the Pi-0 background to be able to monitor both PTI and ETI, considering that AvrBsT induces ETI in the Pi-0 ecotype but not the Col-0 ecotype [22]. A 365-bp hairpin *ACIP* binary construct (hp-ACIP) was designed using the *ACIP1* gene, which included the most conserved region shared by the entire gene family (Figure S7A,B), and then it was transformed into Pi-0 plants. Five independent transgenic RNAi lines were characterized. The hp-ACIP construct significantly reduced the mRNA levels for 4 of the 7 family members (*i.e. ACIP1*, *ACIP-L1*, *ACIP-L2*, and *ACIP-L3*) in two T2 *ACIP* RNAi lines (*i.e.* lines 1 and 29; Figure S7C). Of these 4 genes, *ACIP1* mRNA was the most abundant transcript in 4-week old Pi-0 leaves (Figure S7D), suggesting that it may be the major isoform expressed in leaves.

**Figure 4:**
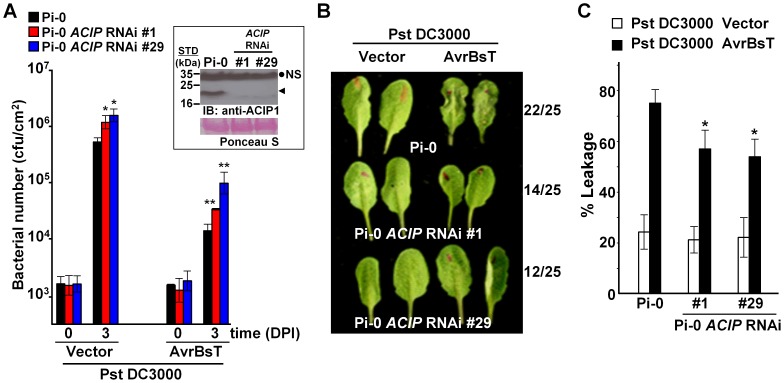
Members of *Arabidopsis* ACIP family are required for AvrBsT-triggered ETI. (A) Increased growth of Pst DC3000 and Pst DC3000 AvrBsT in Pi-0 *ACIP* RNAi line #1 (red bars) and line #29 (blue bars) compared to wild-type Pi-0 (black bars). Leaves were syringe-infiltrated with a 1×10^5^ cells/mL suspension of bacteria. Titers were assessed at 0 and 3 days post-inoculation (DPI). Data are mean cfu/cm^2^ ± SD (n = 4). Asterisks indicate statistically significant differences from Pi-0 (student *t*-test, *p<0.05, **p<0.01). Experiment was repeated three times with similar results. Inset: Immunoblot analysis of protein extracted from Pi-0 and Pi-0 *ACIP* RNAi leaves using anti-ACIP1 sera. Black dot, non-specific band (NS); arrowhead, detected ∼20 kDa protein band expected to correspond to ACIP1, ACIP-L1, and/or ACIP-L3. STD, molecular weight standard in kDa. Ponceau S-stained Rubisco large subunit was used as loading control. (B) AvrBsT-elicited HR phenotype in Pi-0 and Pi-0 *ACIP* RNAi lines. Leaves were infiltrated with a 3×10^8^ cells/mL suspension of Pst DC3000 alone (vector) or Pst DC3000 AvrBsT (AvrBsT). Photograph was taken at 9 hours post-inoculation (HPI). Number of leaves exhibiting confluent HR at 10 HPI out of 25 inoculated leaves is shown at right. (C) Quantification of electrolyte leakage in the leaves described in (B) at 10 HPI. Error bars represent SD (n = 10). Asterisks indicate statistically significant differences from Pi-0 (student's *t*-test, *p<0.05). Experiment was repeated three times with similar results.

To monitor ACIP1 protein expression in leaves, we generated rabbit polyclonal antisera using recombinant ACIP1-His6 protein purified from *E. coli*. The resulting antisera recognized multiple, recombinant purified ACIP isoforms with distinct molecular weights by immunoblot analysis (data not shown). However in wild-type Pi-0 leaf extracts, the antisera only detected a single 20 kDa protein band (Figure 4A, inset). Three of the isoforms have predicted molecular weights in this range: ACIP1 = 20.5 kDa, ACIP-L1 = 20.2 kDa, and ACIP-L3 = 20.9 kDa. The 20 kDa protein band was not detected in the two *ACIP* RNAi lines (Figure 4A, inset) suggesting that ACIP1, ACIP-L1 and/or ACIP-L2 protein accumulation was significantly reduced.

Bacterial growth curves were then performed using a 1×10^5^ cells/mL suspension of Pst DC3000 expressing AvrBsT and the two Pi-0 *ACIP* RNAi transgenic lines to determine if *ACIP* expression is required for AvrBsT-triggered ETI. The phenotypes of the *ACIP*-silenced lines were compared with an unsilenced Pi-0 control plant (Figure 4).

We found that the titer of Pst DC3000 AvrBsT was significantly higher in infected Pi-0 *ACIP* RNAi leaves compared to that in wild-type Pi-0 leaves (Figure 4A). Notably, the Pi-0 *ACIP* RNAi leaves were also more susceptible to Pst DC3000. These data suggested that the silenced ACIP isoforms might function in PTI as well as ETI.

To confirm that AvrBsT-triggered ETI is impaired in the RNAi lines, we performed HR and electrolyte leakage assays in leaves challenged with a high titer (3×10^8^ cells/mL) of Pst DC3000 AvrBsT. ETI in the Pi-0 *ACIP* RNAi lines was delayed but not fully inhibited (Figure 4B). In control Pi-0 leaves, AvrBsT-dependent HR was visible at 9 HPI in many leaves and by 10 HPI, 22/25 leaves exhibited HR. By contrast, HR was not observed in similarly inoculated RNAi leaves at 9 HPI; however, HR started to develop at 10 HPI in 14/25 leaves for line 1 and 12/25 leaves for line 29. Consistent with these findings, electrolyte leakage was significantly reduced in the Pst DC3000 AvrBsT-inoculated Pi-0 *ACIP* RNAi leaves relative to the inoculated Pi-0 leaves at 10 HPI (Figure 4C). These data suggest that multiple ACIP isoforms are required for AvrBsT-triggered ETI symptoms in Pi-0.

The Pi-0 *ACIP* RNAi lines were also examined for their ability to mount ETI in response to two other *Pseudomonas* effectors – AvrB and AvrRpt2 [30], [31]. As observed for AvrBsT, HR symptom development was slower in the RNAi lines infected with a high titer of Pst DC3000 AvrB or Pst DC3000 AvrRpt2 (data not shown). Subsequent bacterial growth curve analyses revealed that the Pi-0 *ACIP* RNAi lines were more susceptible to both Pst DC3000 AvrB and Pst DC3000 AvrRpt2 (Figure S8). These data suggest that the ACIP isoforms play a general role in ETI and are not specific to defense responses elicited by AvrBsT.

**Figure 5:**
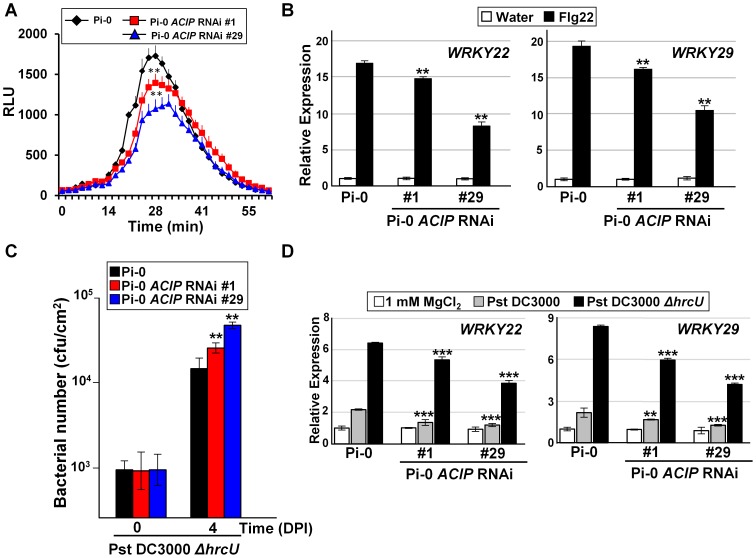
Members of *Arabidopsis* ACIP family are required for PTI. (A) Flg22-stimulated oxidative burst response in Pi-0 and Pi-0 *ACIP* RNAi leaves. RLU = relative luminescence unit. Error bars represent SD (n = 9). Response in both RNAi lines (#1 and #29) was significantly different from that in Pi-0 between time interval 28–32 minutes (student's *t*-test, **p<0.01). (B) Flg22-stimulated PTI marker gene induction in Pi-0 and Pi-0 *ACIP* RNAi leaves. Leaves of three plants were infiltrated with water (control) or 100 nM flg22 and then pooled for RNA extraction. *WRKY22* and *WRKY29* mRNA levels were quantified by qPCR. UBQ5 was used to normalize the expression value for each sample. Relative expression (mean ± SD; n = 4) is shown. (C) Growth of *Pst* DC3000 *ΔhrcU* in Pi-0 (black bars) and Pi-0 *ACIP* RNAi leaves (red and blue bars). Leaves were inoculated with a 1×10^5^ cells/mL suspension of bacteria. Titers were assessed at 0 and 4 DPI. Data are mean cfu/cm^2^ ± SD (n = 4). (D) Pst DC3000 *ΔhrcU*-stimulated PTI marker gene induction in Pi-0 and Pi-0 *ACIP* RNAi lines. Leaves were infiltrated with a 2×10^8^ cells/mL suspension of Pst DC3000 (grey bar), Pst DC3000 (*ΔhrcU*) (black bar) or 1 mM MgCl_2_ (white bar). Samples were collected at 6 HPI and then analyzed as described in (B). Asterisks indicate statistically significant differences from Pi-0 (student's *t*-test,*p<0.05,**p<0.01, ***p<0.001). Similar results were obtained in three independent experiments for (A–C), and two independent experiments for (D).

Given that the RNAi lines were also more susceptible to Pst DC3000, we next examined the potential role of the ACIP family in PTI. First, we analyzed the responsiveness of the Pi-0 *ACIP* RNAi lines to the PAMP elicitor flg22 (Figure 5). Perception of flg22 by the PRR FLS2 results in the production of reactive oxygen species (ROS) [32], one the first measurable PTI responses, followed by changes in PTI gene induction [33]. Flg22-induced ROS production was significantly reduced in both Pi-0 *ACIP* RNAi lines (Figure 5A). Similarly, flg22-induced mRNA accumulation for *WRKY22* and *WRKY29*, two genes encoding transcription factors that positively regulate PTI, was significantly reduced at 3 hr post-treatment in both Pi-0 *ACIP* RNAi lines (Figure 5B). Consistently, the RNAi line 29 exhibited the least responsiveness to flg22 elicitation (Figure 5A,B).

We also examined the responsiveness of the Pi-0 *ACIP* RNAi lines to Pst DC3000 *ΔhrcU*, a *Pseudomonas* strain known to elicit PTI. Pst DC3000 *ΔhrcU* lacks a functional T3S apparatus [34] and does not suppress PTI because T3S effectors are not secreted. Leaves were infected with a 1×10^5^ cells/mL suspension of bacteria and titers were determined at 4 DPI. Pi-0 *ACIP* RNAi leaves were significantly more susceptible to Pst DC3000 Δ*hrcU* (Figure 5C). Consistent with these findings, accumulation of *WRKY22* and *WRKY29* mRNA was significantly reduced at 6 HPI in Pi-0 *ACIP* RNAi leaves compared to wild-type Pi-0 leaves inoculated with a high titer (2×10^8^ cells/mL suspension) of Pst DC3000 *ΔhrcU* (Figure 5D). Taken together, these data suggest that a subset of the ACIP family (*i.e.* ACIP1, ACIP-L1, ACIP-L2, and ACIP-L3) collectively contribute to anti-bacterial immunity in *Arabidopsis*.

### ACIP1 co-localizes with microtubules

**Figure 6:**
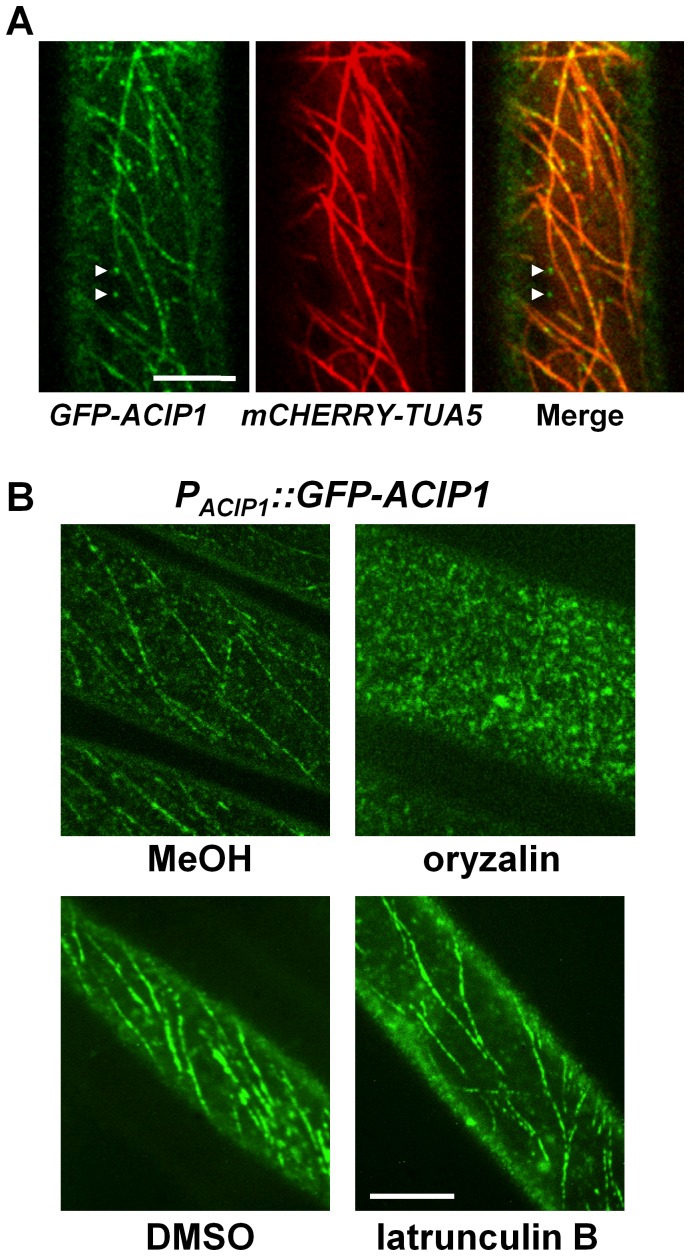
GFP-ACIP1 co-localizes with microtubules. (A) Single plane images of periclinal surface of epidermal cells of 4-day old etiolated Pi-0 *P_ACIP1_*::*GFP-ACIP1/P_35S_::mCHERRY-TUA5* hypocotyl cells. Arrowheads show GFP-ACIP1 punctae that are not associated with microtubules. (B) Localization of GFP-ACIP1 in 4-day old etiolated Pi-0 *P_ACIP1_*::*GFP-ACIP1* hypocotyls treated with MeOH or MeOH +10 µM oryzalin (top panels), or DMSO or DMSO +1 µM latrunculin B (bottom panels). Cells were imaged using confocal microscopy. Bars = 10 µm.

To begin to address ACIP1's function, we examined ACIP1 protein localization in *Arabidopsis* seedlings and mature plants. We generated homozygous transgenic Pi-0 plants expressing a GFP-ACIP1 protein fusion under the control of the native *ACIP1* promoter (*i.e. P_ACIP1_::GFP-ACIP1*). Using confocal microscopy, we observed a low level of GFP-ACIP1 fluorescence in 4-day old etiolated seedlings and juvenile leaves. Little or no detectable fluorescence was observed in mature, senescing leaves. In hypocotyl epidermal cells, GFP-ACIP1 was predominantly found as punctae at the cell cortex. A portion of these punctae was aligned, forming transverse cable-like structures (Figure 6A). ACIP1's subcellular localization pattern partially resembled that of cytoskeletal structures. Unlike TaSRG, the ACIP-L4 ortholog, GFP-ACIP1 was not observed in the plant nucleus, indicating that ACIP1 localization is distinct from this predicted transcription factor [28].

Next, we applied drugs to disrupt the cytoskeleton to determine if ACIP1 co-localizes with actin and/or microtubules. Treatment of the Pi-0 *P_ACIP1_::GFP-ACIP1* seedlings with oryzalin, a microtubule depolymerizing agent, disrupted the GFP-ACIP1 cable-like structures and caused the formation of numerous GFP-ACIP1 punctae throughout the cell (Figure 6B). By contrast, the actin depolymerizing agent latrunculin B did not appear to significantly disrupt these cables (Figure 6B).

To show ACIP co-localization with microtubules, we transformed the Pi-0 *P_ACIP1_::GFP-ACIP1* lines with *P_35S_::mCHERRY-TUA5*, a fluorescently tagged isoform of α-tubulin. A large portion of the GFP-ACIP1 punctae co-localized with mCHERRY-TUA5 microtubules (Figure 6A). Some of the cortical GFP-ACIP1 punctae were not associated with microtubules. Inspection of the literature revealed that ACIP1 was identified in the *Arabidopsis* proteome that co-purified with tubulin by affinity chromatography [27]. We did not detect a direct interaction between ACIP1 and TUA5 in a targeted yeast two-hybrid assay (Figure S5A,B). It is possible that ACIP1 association with tubulin might be indirect or via a weak electrostatic interaction. Or, ACIP1 might interact with another isoform of tubulin. Collectively, our findings indicate that GFP-ACIP1 signal forms punctae on the cell cortex and some of these punctae co-localize with the cortical microtubule network.

### AvrBsT alters ACIP1 localization

**Figure 7:**
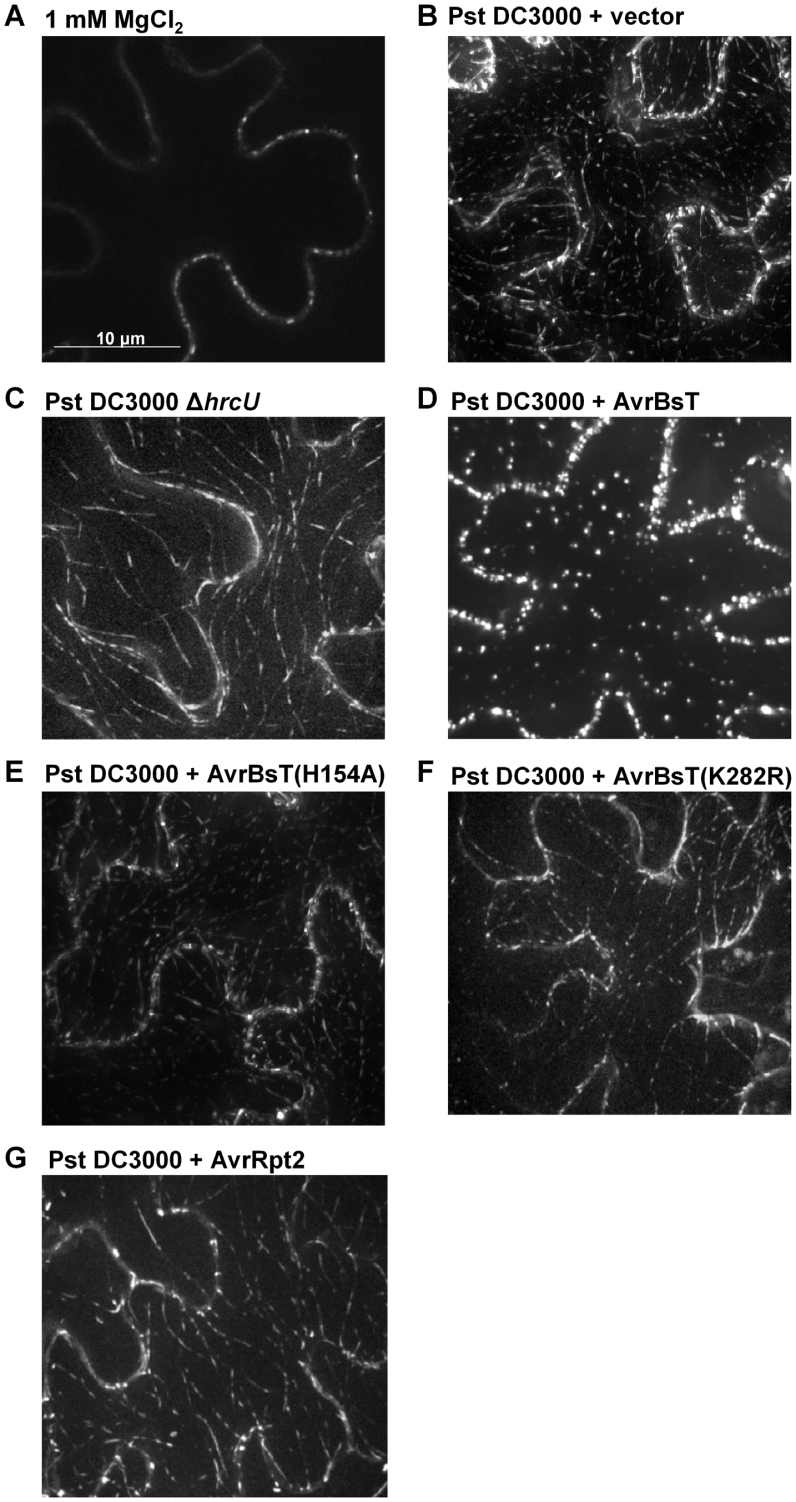
AvrBsT alters GFP-ACIP1's localization in a catalytic-dependent manner. Pi-0 *P_ACIP1_*::*GFP-ACIP1* leaves were inoculated with (A) 1 mM MgCl_2_ or a 3×10^8^ cells/mL suspension of (B) Pst DC3000 vector, (C) Pst DC3000 Δ*hrcU*, (D) Pst DC3000 AvrBsT, (E) Pst DC3000 AvrBsT(H154A), (F) Pst DC3000 AvrBsT(K282R), or (G) Pst DC3000 AvrRpt2. Spinning disk confocal images were recorded at 6–7 HPI. Bar = 10 µm. Similar results were obtained in more than 3 independent experiments.

We speculated that AvrBsT-binding to and acetylation of ACIP1 might interfere with ACIP1's stability and/or localization within plant leaves during pathogen infection. We did not detect by protein gel blot analysis any differences in endogenous ACIP1 protein abundance or mobility using extracts isolated from Pi-0 leaves infected with Pst DC3000 or Pst DC3000 AvrBsT (Figure S6B). However, we did notice that GFP-ACIP1 localization in 4-week old Pi-0 *P_ACIP1_::GFP-ACIP1* leaves was dramatically altered in response to both Pst DC3000 and Pst DC3000 AvrBsT (Figure 7). Unlike the signal in young hypocotyls, GFP-ACIP1 fluorescence at the cortex of epidermal cells in 4-week old leaves inoculated with the 1 mM MgCl_2_ mock control was diffuse and faint. This signal was difficult to capture in the image projection and varied among plants. By contrast, GFP-ACIP1 punctae were observed at or near the cell periphery of these cells (Figure 7A). Infection with Pst DC3000 for 6 h led to the formation of rod-shaped GFP-ACIP1 structures of various lengths (Figure 7B), which were difficult to detect in the 1 mM MgCl_2_ mock control (Figure 7A). The GFP-ACIP1 rods were also detected in response to Pst DC3000 *Δ*hrcU (Figure 7C), indicating that these structures are associated with PTI in a T3S effector-independent manner. Strikingly, Pst DC3000 AvrBsT infection for 6 h led to the formation of large, bright GFP-ACIP1 aggregates and fewer rod-like structures (Figure 7D). This localization pattern was dependent on AvrBsT's catalytic activity. Pst DC3000 AvrBsT (H154A) infection resulted in a GFP-ACIP1 signal (Figure 7E) similar to that induced by Pst DC3000 alone (Figure 7B). Similarly, Pst DC3000 AvrBsT(K282R) infection led to the formation of GFP-ACIP1 rods (Figure 7F), but not large aggregates.

To determine if GFP-ACIP1 aggregates are generated specifically by AvrBsT and/or in response to PA production, we tested the phenotype of Pst DC3000 AvrRpt2. AvrRpt2 elicits ETI in Pi-0 leaves [23], which is dependent on PA production [23], [35]. Infection with Pst DC3000 AvrRpt2 induced the formation of GFP-ACIP1 rods (Figure 7G), similar to those formed in response to Pst DC3000 alone, Pst DC3000 AvrBsT(H154A), and Pst DC3000 AvrBsT(K282R) (Figure 7B,E,F). However, ACIP1 aggregates were not observed in response to Pst DC3000 AvrRpt2. Pi-0 *P_ACIP1_::GFP-ACIP1* leaves were inoculated with 50 µM PA and then imaged the leaves 1.5 hr later. Exogenous PA triggered the formation of several GFP-ACIP1 rods but only a few punctae (Figure S8C), whereas the mock control containing 0.2% DMSO did not (Figure S8D). These data suggest that PA exposure is sufficient to promote the formation of ACIP1 punctae and rods, but not the formation of ACIP1 aggregates. Moreover, they indicate that ACIP1 aggregate formation is a specific phenotype linked to AvrBsT acetyltransferase activity *in planta*.

## Discussion

Pathogen-dependent acetylation of host targets has emerged as a key virulence strategy to alter eukaryotic defense responses. The study of several YopJ and YopJ-like effectors in animals and flies indicates that O-acetylation of Ser/Thr residues or Nε-acetylation of Lys residues in the activation loop of kinases in innate immune pathways directly interferes with residue phosphorylation or ATP binding, respectively [6]–[8], [18]. Both scenarios prevent the activation of kinases that are required to mediate innate immune signal transduction. In plants, the mechanism(s) by which YopJ-like effector acetylation of host substrates modulates immune signaling is less clear.

Based on this study, we propose that the YopJ-like effector AvrBsT acetylates *Arabidopsis* ACIP1. The role of ACIP1 *in planta* is not known; however, it is predicted to be a small α-helical protein [25]. ACIP1 emerged from an *Arabidopsis* screen looking for tubulin-binding proteins [27], suggesting that it might be a microtubule-associated protein. Our microscopy studies of *Arabidopsis* Pi-0 lines expressing GFP-ACIP1 revealed that ACIP1 is localized to punctae on the cell cortex and some of these punctae co-localize with the cortical microtubule network (Figure 6). These data are consistent with ACIP1 being a part of the tubulin proteome [27].

Importantly, we discovered that GFP-ACIP1 organization and accumulation changed significantly during bacterial infection (Figure 7). Numerous small GFP-ACIP1 punctae and rod-like structures formed throughout the cell in response to Pst DC3000 infection. These structures also formed during Pst DC3000 *ΔhrcU* infection, indicating that changes in ACIP1 localization are coincident with PTI. Strikingly, Pst DC3000 AvrBsT infection, but not Pst DC3000 AvrRpt2 infection, dramatically altered GFP-ACIP1 localization. AvrBsT activity triggered the accumulation of large GFP-ACIP1 aggregates throughout the plant cell. The aggregates did not appear to be aligned like those observed in leaves infected with Pst DC3000 or uninfected hypocotyl epidermal cells (Figure 6). Interestingly, a mutation (K282R) that disrupted AvrBsT's ability to acetylate ACIP1 *in vitro* (Figure 2B) also prevented the formation of GFP-ACIP1 aggregates (Figure 7) and activation of ETI during Pst DC3000 AvrBsT infection (Figure 3). Taken together, these data suggest the model that AvrBsT acetyltransferase activity *in planta* uniquely alters ACIP1's localization, which is linked to AvrBsT-dependent activation of ETI.

The nature of the large GFP-ACIP1 aggregates and their function during pathogen infection remains to be determined. Given the requirement for ACIP1 for both PTI (Figure 5) and the formation of ACIP1 punctae and rods during Pst DC3000 infection (Figure 7), we speculate that ACIP1 association with microtubules and/or the cell cortex is important for plant immunity. We also speculate that AvrBsT acetyltransferase activity either directly or indirectly alters ACIP1 association with microtubules. Association of ACIP1 with microtubules may play a direct role in microtubule organization, or it may be involved in microtubule-dependent processes such as vesicle and protein trafficking. Alternatively, ACIP1 may simply use microtubules to position itself and its interacting proteins at the cell cortex, where plant cells first encounter injected bacterial effectors. Our *ACIP* RNAi plants (silenced for *ACIP1, ACIP-L1, ACIP-L2*, and *ACIP-L3*, Figure S7) did not show cell shape or cell growth phenotypes, which are caused by microtubule cytoskeleton defects, suggesting that four ACIP family members do not regulate microtubule cytoskeleton structure. Future functional studies will test if ACIP1 and/or other isoforms expressed in leaves play a role in suppressing bacterial growth by regulating microtubule-dependent trafficking or by regulating other processes at the plasma membrane or cell cortex.

Notably, AvrBsT catalysis in *Arabidopsis* Pi-0 leaves leads to the accumulation of PA, a lipid signal associated with plant adaptation to biotic and abiotic stress [24]. Elevated PA levels in Pi-0 leaves inoculated with Pst DC3000 AvrBsT correlate with ETI [22], [23]. ACIP1 is required for AvrBsT-triggered ETI (Figure 4); however, the causal relationship between changes in PA production and ACIP1 localization in response to AvrBsT acetyltransferase activity is not clear. Furthermore, it is not known if PA is required to alter ACIP1 localization and/or function. Exogenous PA treatment (Figure S8C) or infection with Pst DC3000 AvrRpt2 (Figure 7G), which triggers a PA burst during ETI [35], induced the formation of small ACIP1 punctae and rods of various sizes, but large ACIP1 aggregates similar to those formed in response to AvrBsT-dependent catalysis (Figure 7D) were not observed. These data further highlight the specificity for AvrBsT in inducing ACIP1 aggregation during infection. They also suggest that a threshold concentration of PA or local production of PA relative to ACIP1 might be required to trigger the formation of large ACIP1 aggregates *in planta*.

PA is known to play a critical role in the regulation of cytoskeletal dynamics [36]. Recent data suggests that PA alters the microtubule network by directly binding to cytoskeletal components, including tubulin and the microtubule bundling protein MAP65-1 [37], [38]. Interestingly, elevated PA resulting from salt stress recruits *Arabidopsis* MAP65-1 to the membrane and enhances its ability to stabilize microtubules, which promotes cell survival [38]. How PA directly alters the microtubule network during ETI is not known. The link between PA, ACIP1, and the microtubule network during pathogen infection established in this study suggests that PA might regulate ACIP1 complex formation and/or association with microtubules.

Interestingly, the HopZ1a acetyltransferase was recently shown to disrupt plant cortical microtubule arrays and secretion during bacterial infection [10]. In this case, *P. syringae* HopZ1a infection led to reduced microtubule density, suggesting that HopZ1a acetylation *in planta* affects the stability or nucleation of microtubules. Acetylation of mammalian EB1, a microtubule-associated protein, which promotes microtubule assembly, was recently shown to compromise EB1 binding to other microtubule plus-end tracking proteins [39]. HopZ1a binds and acetylates tubulin *in vitro*. Whether or not HopZ1a modifies tubulin and/or affects microtubule properties (*i.e.* assembly, disassembly, and/or stability) during infection remains to be determined.

In terms of acetylation, our data suggests that AvrBsT trans-acetylation activity, not auto-acetylation activity, triggers ETI in Pi-0 leaves (Figure 3). Mutation of Lys 282 to Arg in AvrBsT, a conserved residue found in YopJ and YopJ-like effectors [9], did not affect AvrBsT's auto-acetyltransferase activity *in vitro* (Figure 2A), although it inhibited its ability to trans-acetylate ACIP1 (Figure 2B). We speculate that K282 is required for enzyme-substrate interactions, although acetyl-CoA docking or direct acetylation [9] is also possible. Importantly, Pst DC3000 expressing the AvrBsT(K282R) mutant failed to trigger ACIP1 aggregates (Figure 7) and elicit host resistance (Figure 3). These data suggest that acetylation is linked to changes in ACIP1 function and immunity. Whether or not acetylation of ACIP1 is directly linked to punctae formation, localization with microtubules, PA production and/or the activation of AvrBsT-triggered ETI awaits characterization of ACIP1's acetylation status *in planta*. Since acetylation can increase the electronegativity of proteins, it has the potential to disrupt ACIP1 interactions with the negatively charged microtubule lattice. Electrostatic interactions have been shown to play a significant role in microtubule binding of motor proteins and microtubule-associated proteins that often possess domains enriched in positively charged residues [40]–[42]. Future mapping of the ACIP1 microtubule-interaction domain in relation to residues acetylated by AvrBsT will allow us to test the functional significance of ACIP1 acetylation *in planta*.

A growing number of plant targets have been identified for YopJ-like effectors, questioning the specificity of these enzymes as acetyltransferases versus binding partners in immune complexes. Our work indicates that there is selectivity between AvrBsT and HopZ1a *in vitro*. AvrBsT acetylates ACIP1 whereas HopZ1a acetylates tubulin. In addition to ACIP1, AvrBsT has been recently shown to bind to arginine decarboxylase (ADC1), an enzyme proposed to mediate polyamine and γ-aminobutyric acid metabolism and impact cell death responses [43], and SNF-1 related kinase (SnRK1), a putative regulator of sugar metabolism [44]. Post-translational acetylation of these plant proteins has not yet been reported. The fact that a number of metabolic enzymes are normally regulated by acetylation warrants further investigation [45].

Similarly, HopZ1a appears to have multiple plant targets. In addition to tubulin, HopZ1a was shown to acetylate *Arabidopsis* ZED1, a pseudokinase required for HopZ1a-dependent ETI [17], and *Arabidopsis* jasmonate (JA) ZIM-domain proteins required to repress JA signaling during PTI [46]. HopZ1a was also shown to bind and destabilize an enzyme involved in isoflavonoid biosynthesis, 2-hydroxyisoflavanone dehydratase (HID1), by an unknown mechanism [47]. The diverse nature of these targets suggests that HopZ1a is a promiscuous enzyme capable of altering defense signaling at multiple nodes.

It is intriguing that HopZ2, the closest YopJ-like homolog to AvrBsT [48], was found to directly interact with *Arabidopsis* MLO2 *in planta* [49]. *Arabidopsis mlo2-7* mutants are compromised for HopZ2-dependent virulence, further supporting the role of MLO2 as a negative regulator of immunity [49]. MLO2 is a plasma membrane protein of unknown function that interferes with vesicular trafficking mediated by the syntaxin PEN1 [50], [51]. It is too early to tell if there is a common theme for YopJ-like targets in plant cells. However, the identification of ACIP1, tubulin, and MLO2 as host targets suggests that some YopJ-like effectors might have undergone specialization to interfere with the trafficking function of the microtubule cytoskeleton in infected cells.

Does AvrBsT target ACIP1 or an ACIP1 complex to suppress immunity? This question has been difficult to answer because we have yet to detect an AvrBsT virulence phenotype in *Arabidopsis* during bacterial infection. This is not so surprising given the potential functional redundancy between AvrBsT and the suite of T3S effectors in Pst DC3000. Overexpression of AvrBsT in transgenic *Arabidopsis* lines however was recently shown to enhance susceptibility to Pst DC3000 [52]. In solanaceous plants, AvrBsT is known to suppress PTI in tomato [53] and ETI in pepper [44] during *Xanthomonas* infection. The study of the ACIP1 ortholog in tomato may provide insight to AvrBsT virulence, by specifically addressing how AvrBsT acetyltransferase activity interferes with ACIP1's function during PTI and/or ETI.

In summary, the study of AvrBsT-triggered defense responses in *Arabidopsis* Pi-0 plants has led to the identification of ACIP1, a member of a new protein family required for PTI and ETI. We demonstrate that the expression of four *Arabidopsis* ACIP isoforms (ACIP1, ACIP-L1, ACIP-L2, and ACIP-L3) is required for proper execution of PTI in response to Pst DC3000 (Figure 5) and ETI in response to Pst DC3000 expressing AvrBsT, AvrB or AvrRpt2 (Figure 4, S8). In addition, we show that AvrBsT is an acetyltransferase and provide evidence that acetyltransferase activity plays an important role in altering ACIP1 localization within the plant cell during infection and the activation of ETI. This study highlights an important link between ACIP1 and the microtubule network during plant defense.

## Materials and Methods

### Bacterial strains and growth

*Escherichia coli* DH5 alpha and *Agrobacterium tumefaciens* strain GV3101 were grown on Luria agar medium at 37 and 28°C, respectively. *Pseudomonas syringae* pathovar *tomato* (Pst) DC3000 strains were grown on nutrient yeast glycerol agar (NYGA) [54] at 28°C. *E. coli* antibiotic selection was 100 µg/mL carbenicillin and/or 50 µg/mL kanamycin. *A. tumefaciens* antibiotic selection was 100 µg/mL rifampicin, 50 µg/mL kanamycin, and/or 30 µg/mL gentamicin. Pst antibiotic selection was 100 µg/mL rifampicin, and/or 50 µg/mL kanamycin.

### Plant lines and growth

*Arabidopsis thaliana* Col-0 and Pi-0 ecotypes were grown in growth chambers (22°C, 60% RH, 125 µE/m^2^/s fluorescent illumination) on an 8-h light/16-h dark cycle. Plants were transformed using the floral dip method [55].

### Molecular constructions

Standard DNA cloning methods [56], PCR, and Gateway technology (Invitrogen) were used for plasmid construction. All primer sequences are listed in Table S1. For GST-AvrBsT, *avrBsT* (wild type, H154A, C222A, and K282R) was amplified by PCR, cloned into pJET1.2, and then sub-cloned into pGEX-5X-3 using *Bam*HI and *Xho*I. For Gateway constructions, amplified PCR products (*i.e. avrBsT*, *hopZ1a*, *hopZ1b*, *hopZ2*, *hopZ3*, *ACIP1*, *ACIP-like* genes (*ACIP-L1* to *ACIP-L6*), and *TUA5*) were cloned into pCR8 to create donor plasmids. The respective donor plasmids were recombined into: 1) pGADT7 to create AD-gene fusions and pGBKT7 or pXDGATcy86 to create BD-gene fusions for two-hybrid analysis; 2) pDEST15 for GST-fusions; and/or 3) pDEST17 for His6-fusions. For *avrBsT* mutagenesis, QuikChange Site –Directed Mutagenesis kit (Stratagene) was performed with pCR8(*avrBsT*) and PfuUltra II Fusion HS DNA polymerase (Agilent).

### Yeast two-hybrid screen

Yeast strain AH109 carrying pXDGATcy86(*avrBsT*) was transformed with pAD-GAL4-2.1 containing the Horwitz and MA cDNA library isolated from *A. thaliana* inflorescence meristem, floral meristem, and floral buds (obtained from TAIR). Approximately 7 million transformants were screened and interaction with At3g09980 cDNA was confirmed.

### Yeast protein extraction

Yeast cells were resuspended in lysis buffer (1.85 M NaOH and 7% 2-mercaptoethanol) and then proteins were precipitated in 10% trichloroacetic acid. Protein pellets were washed in 1 M Tris and then resuspended in 8 M urea sample buffer.

### Protein gel blot analysis

Protein was extracted from plant cells as described [34], separated by SDS-PAGE, transferred to nitrocellulose, and then detected by ECL or ECL plus (GE Healthcare) using anti-ACIP1, anti-HA (Covance), anti-Myc (Covance), anti-His (Qiagen), or anti-GST (Santa Cruz) sera and horseradish peroxidase conjugated secondary antibodies (Bio-Rad). Membranes were stained with Ponceau S to control for loading.

### ACIP1 antibody production

Recombinant His6-ACIP1 was expressed in *E. coli* BL21 tRNA cells and purified using Ni-NTA agarose under denaturing conditions following manufacturer's protocol (Qiagen). Polyclonal antisera were raised in rabbits using the purified His-ACIP1 protein (Covance).

### *In vitro* GST pull-down assay

GST or GST-AvrBsT were expressed in *E. coli* BL21-CodonPlus(DE3) cells (Stratagene). Cells were lysed in buffer (1X PBS, pH 8, 1% Triton X-100, 0.1% 2-mercaptoethanol, and 1 mM phenylmethylsulfonyl fluoride (Sigma Aldrich)) with a sonicator (Branson). GST and GST-AvrBsT supernatants were incubated with 30 µL of pre-equilibrated Glutathione Sepharose 4B (GE Healthcare) for 1 h at 4°C with rotation. Sepharose beads were recovered by centrifugation and then washed with buffer for 5 min at 4°C with rotation. GST or GST-AvrBsT (WT, C222A, or K282R) bound beads were incubated with soluble *E. coli* lysates containing His6-ACIP1 for 2 h at 4°C with rotation. The beads were washed with buffer (50 mM TrisHCl, pH 7.5, 150 mM NaCl, 10 mM MgCl_2_, 0.1% Triton X-100, and 0.1% 2-mercaptoethanol) three times. Protein bound to the beads was separated by SDS-PAGE and analyzed by immunoblot analysis. Anti-GST and anti-His sera were used to detect GST-AvrBsT and His6-ACIP1.

### Protein acetylation assay

Purified recombinant GST-tagged proteins (1 µg each) were incubated with 100 nM inositol hexakisphosphate (IP_6_) (Santa Cruz), 0.4 µCi ^14^C-acetyl-CoA (Perkin Elmer) in 50 mM TrisHCl pH 8 and 1 mM DTT for 30 min at RT. Urea sample buffer was added to stop the reactions. Proteins assayed included: GST, GST-AvrBsT, GST-AvrBsT(C222A), GST-AvrBsT(H154A), GST-AvrBsT(K282R), GST-HopZ1a, and GST-ACIP1. Proteins were separated in a 10% SDS-PAGE gel, stained with Coomassie blue, transferred to blotting paper, dried, treated with EN3HANCE (Perkin Elmer), and then exposed to film for 2–3 weeks at 80°C.

### *ACIP* silencing construct and transgenic *Arabidopsis* plants

A 365 bp region of *ACIP1* was PCR amplified using primer set JG616/JG617, and the product was cloned into pKANNIBAL to create pKANNIBAL(hp-*ACIP*). The *Not*I fragment was then subcloned into pART27 [57], creating pAR27(hp*-ACIP*). The resulting plasmid was then transformed into *A. thaliana* ecotypes Col-0 and Pi-0. Transformants were analyzed by quantitative RT-PCR to measure *ACIP* isoform mRNA levels using primer sets listed in Table S1.

### Bacterial HR and growth assays

Fully expanded leaves of 4- to 5-week-old plants were used for bacterial inoculations. A suspension of bacterial cells (Pst DC3000, Pst DC3000 AvrBsT, Pst DC3000 AvrB, Pst DC3000 AvrRpt2, or Pst DC3000 *ΔhrcU*; 3×10^8^ cells/mL for HR and 1×10^5^ cells/mL for growth curves) was infiltrated into the extracellular space of fully expanded leaves using a 1-mL syringe. For HR, plants were incubated at RT under lights and phenotypes were recorded 9–12 HPI. For growth curves, plants were incubated at high humidity in a growth chamber for 4 d. Leaf tissue was collected at 0–4 DPI, ground in 1 mM MgCl_2_, diluted and then plated on NYGA plates containing appropriate antibiotics and cycloheximide (50 µg/mL) in triplicate to determine bacterial load. Four plants were used and the experiment was repeated at least three times. The average bacterial titer ± SD is reported.

### Electrolyte leakage assay

Three fully expanded leaves of 4- to 5-week-old plants (n = 4) were inoculated with a 3×10^8^ cells/mL suspension of Pst DC3000 (vector) or Pst DC3000 (AvrBsT). Ten HPI, three leaf discs (10 mm diameter) per plant were floated in 20 mL of water in petri dishes for 5 min and then transferred to a test tube containing 3 mL of water. Tubes were incubated for 1 h at RT with shaking. Conductivity of the solution was measured with an EC meter (Spectrum Technologies) before and after boiling for 30 min [58]. Percent electrolyte leakage was calculated as conductivity before boiling/conductivity after boiling ×100. Assay was repeated at least three times.

### Oxidative burst assay

Three leaf discs (5 mm diameter) from the youngest fully expanded leaves of a 4-week-old plant (n = 9–18) were incubated in water in a 96-well plate (one leaf disc per well) for 24 h. To measure ROS, leaf discs were treated with ± flg22 (100 nM) in 10 µg/mL horseradish peroxidase and 100 µM Luminol (Sigma), and then luminescence was immediately measured with a 1420 Multilabel Counter (PerkinElmer) [32]. Relative luminescence units (RLU) are reported. Assay was repeated at least three times.

### RNA isolation and quantitative RT-PCR

Total RNA was isolated from uninfected or infected leaves using Trizol reagent (Invitrogen) according to manufacturer's instructions. For infection, leaves were inoculated with 1 mM MgCl_2_ or bacterial strains (2×10^8^ cells/mL in 1 mM MgCl_2_) and then one leaf from three plants was harvested, pooled, and total RNA was extracted. 2.5 µg of RNA were used for cDNA synthesis. Quantitative RT-PCR was performed using the cDNA and gene-specific primers (Table S1). Each cDNA was amplified by real-time PCR using SensiFAST SYBR Kit (Bioline) and the MJ Opticon 2 instrument (Bio-Rad). *UBQ5* or *ACTIN8* expression was used to normalize the expression value in each sample and relative expression values were determined against the average value of buffer or bacterially infected sample using the comparative Ct method (2^−ΔΔCt^).

### Microscopy

To monitor ACIP1 protein expression and localization, the promoter region (1.5-kb upstream of start) was fused with GFP-ACIP1 in the backbone of pMDC43 to create pMDC43(*P_ACIP1_::GFP-ACIP1*). The resulting plasmid was transformed into *A. thaliana* Pi-0 plants. Transgenic *P_ACIP1_::GFP-ACIP1* lines were then transformed with P_35S_::mCHERRY-HA-TUA5. This plasmid was construct by modifying pEarleygate 104 [59]. *YFP* was substituted with *mCHERRY* and *Arabidopsis TUA5* genomic coding region was inserted after *mCHERRY*. Localization of GFP-ACIP1 and mCHERRY-TUA5 in 5-day old dark grown hypocotyls was determined using a Leica TCS SP5 confocal microscope (Leica Microsystems) with Leica LAS AF software and a Leica spinning disc confocal microscope with the Yokogawa CSUX-M1 confocal scanner. Seedlings were treated with 10 µM oryzalin in MeOH for 8 hr at RT or 1 µM latrunculin B in DMSO for 4 hr at RT and then imaged. For infection, Pi-0 *P_ACIP1_::GFP-ACIP1* leaves were inoculated with 1 mM MgCl_2_ or bacterial strains (3×10^8^ cells/mL in 1 mM MgCl_2_) for 6 h. For exogenous PA treatment, Pi-0 *P_ACIP1_::GFP-ACIP1* leaves were inoculated with 50 µM PA in 0.2% DMSO or 0.2% DMSO. Images were analyzed using ImageJ [60].

### Accession numbers

Sequence data from this article can be found in the Arabidopsis Genome Initiative or GenBank/EMBL databases under the following accession numbers: At3G09980 (ACIP1), At5G03660 (ACIP-L1), At2G36410 (ACIP-L2), At3G52920 (ACIP-L3), At2G27740 (ACIP-L4), At3G52900 (ACIP-L5) and At2G36355 (ACIP-L6).

## Supporting Information

Figure S1 ***Arabidopsis*** **ACIP protein family.** (A) Amino acid sequence alignment of ACIP1 and six ACIP-like isoforms from *Arabidopsis* using Clustal W. (B) Tree of *Arabidopsis* ACIP protein family was generated by Neighbor-Joining method with default option (1000 bootstrap replicates and complete deletion for gaps/missing data) using MEGA4 software [61]. Bootstrap values are indicated in each branch and the bars represent branch lengths equivalent to 0.05 amino acid changes per residue. *Arabidopsis* gene numbers are listed next to assigned protein names. (C) Yeast two-hybrid assay showing AvrBsT binding to ACIP1 and ACIP-like isoforms. Yeast strains analyzed were AH109 carrying pXDGATcy86 (vector or AvrBsT) BD) and pGADT7(vector, ACIP1, ACIP-L1, ACIP-L2, ACIP-L3, ACIP-L4, ACIP-L5, or ACIP-L6). (D) Immunoblot analysis of proteins isolated from the yeast cells described in Figure 1A and Figure S1C. Analysis was performed using anti-HA sera to detect AD-HA or AD-HA-ACIP fusions and anti-GAL4DBD sera to detect BD or BD-AvrBsT. STD, molecular weight standard in kDa. Expected molecular weights: AD-HA-ACIP fusions = ∼38–41 kDa; BD = 16 kDa; BD-AvrBsT = 55 kDa.
(TIF)

Figure S2 **AvrBsT acetyltransferase activity is stimulated by inositol hexakisphosphate (IP_6_).** Purified GST (negative control), GST-HOPZ1a (positive control), GST-AvrBsT, and GST-AvrBsT(C222A) proteins were incubated with 0.4 µCi ^14^C-acetyl CoA ±100 nM IP_6_ for 30 min at room temperature. Proteins were separated by 10% SDS-PAGE. Protein gel was stained with Coomassie and then analyzed by autoradiography. Acetylated proteins (GST-HopZ1a-^AC^ and GST-AvrBsT-^AC^) are labeled in the autoradiograph. STD, molecular weight standard in kDa. GST = 28 kDa; GST-HopZ1a = 70 kDa; GST-AvrBsT = 65 kDa.
(TIF)

Figure S3 **AvrBsT K282R mutant has auto-acetylation activity** ***in vitro*** **but exhibits reduced HR symptoms** ***in planta*** **.** (A) Alignment of the conserved K282 residue (*) in AvrBsT with other homologs in the YopJ effector family: YopJ from *Yersinia pestis*, PopP2 from *Ralstonia solanacearum*, AvrXv4 and XopJ from *Xanthomonas euvesicatoria*, and HopZ1a, HopZ2, and HopZ3 from *Pseudomonas syringae*. (B) Auto-acetylation activity of AvrBsT(K282R) relative to wild type AvrBsT. Purified GST-AvrBsT(K282R) or GST-AvrBsT (0.5 to 5 µg) was incubated with 0.4 µCi ^14^C -Acetyl CoA and 100 nM IP_6_ for 1 hr at room temperature. GST-AvrBsT(C222A) and GST-AvrBsT(H154A) (5 µg) were used as negative controls. Proteins were separated by 10% SDS-PAGE. Gels were stained with Coomassie and then analyzed by autoradiography. STD, molecular weight standard in kDa. GST-AvrBsT = 65 kDa. (C) Wild type and mutant AvrBsT protein expression level in the *Arabidopsis* Pi-0 leaves described in Figure 2C. Proteins were detected by immunoblot analysis using HA sera. Ponceau S staining was used to detect Rubisco, which served as a loading control.
(TIF)

Figure S4 **AvrBsT mutants interact with ACIP1** ***in vitro*** **.** GST-AvrBsT mutant protein affinity purification of His-ACIP1 *in vitro*. GST, GST-AvrBsT (WT), GST-AvrBsT(C222A) (C/A), or GST-AvrBsT(K282R) (K/R) was incubated with *E. coli* extracts containing His6-ACIP1. Proteins were purified by using glutathione sepharose and analyzed by immunoblot (IB) analysis using anti-GST and anti-His sera. Protein input is shown on left and pull-down on right. Expected protein MW = GST = 28 kDa; GST-AvrBsT mutants = 65 kDa; and His6-ACIP1 = 28 kDa. +, protein expressed,; −, vector control. STD, molecular weight standard. Similar phenotypes were observed in two independent experiments.
(TIF)

Figure S5 **HopZ1a does not interact with or acetylate ACIP1** ***in vitro*** **.** (A) Candidate interaction test using yeast cells carrying AvrBsT, HopZ1a, HopZ1b, HopZ2, HopZ3, or TUA5 in pXDGATcy86(BD) and ACIP1 or TUA5 in pGADT7(AD). Strains were spotted on nonselective (SD –LW) and selective (SD –LWH ±2 mM 3-AT) media and then incubated at 30°C for 3 d. (B) Immunoblot analysis of proteins isolated from the yeast cells described in (A). Anti-HA and anti-GAL4DBD sera were used to detect proteins in pGADT7 and pXDGATcy86, respectively. Expected molecular weights: AD-vector = 23 kDa; AD-AvrBsT = 58 kDa; AD-HopZ1a = 63 kDa; AD-HopZ1b = 63.5 kDa; AD-HopZ2 = 60.5 kDa; AD-HopZ3 = 68.1 kDa; AD-TUA5 = 78 kDa; AD-ACIP1 = 43 kDa; BD = 16 kDa; BD-ACIP1 = 36 kDa; BD-TUA5 = 71 kDa. (C) HopZ1a acetylates tubulin but not ACIP1. Purified GST, GST-HopZ1a, GST-AvrBsT, or GST-AvrBsT(C222A) was incubated with Porcine tubulin (Cytoskeleton), GST, or GST-ACIP1 with 0.4 µCi ^14^C -Acetyl CoA and 100 nM IP_6_ for 1 hr at room temperature. Protein gels were stained with Coomassie and then analyzed by autoradiography. Acetylated proteins are labeled in the autoradiograph. GST = 28 kDa; GST-HopZ1a = 70 kDa; GST-AvrBsT = 65 kDa; tubulin = 55 kDa; GST-ACIP1 = 50 kDa. STD, molecular weight standard in kDa.
(TIF)

Figure S6 **Levels of ACIP1 mRNA and protein abundance in** ***Arabidopsis*** **leaves during Pst DC3000 infection.** Fully expanded Pi-0 leaves (n = 3 plants) were infiltrated with 1 mM MgCl_2_ (control) or a 2×10^8^ cells/mL suspension of Pst DC3000 alone (vector) or Pst DC3000 expressing AvrBsT (AvrBsT). (A) Relative expression of ACIP1 transcript at 3 and 6 HPI. Data were normalized using Actin8. Error bars represent ± SD. This experiment was repeated twice with similar results. (B) Immunoblot analysis of proteins isolated from two plants (1 and 2) inoculated with the strains described above. Anti-ACIP1 sera were used to detect endogenous protein expression. Ponceau S stained Rubisco large subunit was used as a loading control.
(TIF)

Figure S7 **Transcript levels in** ***Arabidopsis ACIP*** **RNAi lines.** (A) Alignment of the nucleotide sequence for *ACIP* gene family in *Arabidopsis*. Primers JG616 and JG617 were used to construct the hairpin *ACIP* RNAi construct in pKannibal (B) which was used to generate pART27(hp-*ACIP*). (C) *ACIP1* and *ACIP-like* mRNA levels in *Arabidopsis* Pi-0 and Pi-0 *ACIP* RNAi lines #1 and #29. (D) Relative expression of *ACIP1* and *ACIP-like* mRNAs in 4-week old, fully expanded leaves determined by qRT-PCR. For C and D, gene specific primers were used (Table S1). Data were normalized using UBQ5. Error bars represent ± SD. This experiment was repeated twice with similar results.
(TIF)

Figure S8 **Members of** ***Arabidopsis ACIP*** **gene family are required for AvrB or AvrRpt2-triggered ETI.** Increased growth of Pst DC3000, Pst DC3000 AvrB, or Pst DC3000 AvrRpt2 in Pi-0 *ACIP* RNAi line #1 (red bars) and line #29 (blue bars) compared to wild-type Pi-0 (black bars). Leaves were syringe-infiltrated with a 1×10^5^ cells/mL suspension of bacteria. Titers were assessed at 0 and 3 days post-inoculation (DPI). Data are mean cfu/cm^2^ ± SD (n = 4). Asterisks indicate statistically significant differences from Pi-0 (student *t*-test, **p<0.01). Experiment was repeated twice with similar results.
(TIF)

Figure S9 **GFP-ACIP1 localization changes in response to different treatments.** Pi-0 *P_ACIP1_*::*GFP-ACIP1* leaves were inoculated with: (A) 1 mM MgCl_2_, or a 3×10^8^ cells/mL suspension of (B) Pst DC3000 AvrBsT, (C) 50 µM PA in 0.2% DMSO or (D) 0.2% DMSO. Spinning disk confocal images were recorded at 6–7 HPI (A–B) or 1.5 HPI (C–D). Bar = 10 µm. Similar results were obtained in more than 3 independent experiments.
(TIF)

Table S1 **Primer sequences used in this study, related to Experimental Procedures.**
(DOCX)
