# Supplementary figures and images for: AvrBsT Acetylates Arabidopsis ACIP1, a Protein that Associates with Microtubules and Is Required for Immunity
Source: PLoS Pathog. 2014 Feb 20;10(2):e1003952. doi: 10.1371/journal.ppat.1003952 (PMC3930583; doi:10.1371/journal.ppat.1003952)

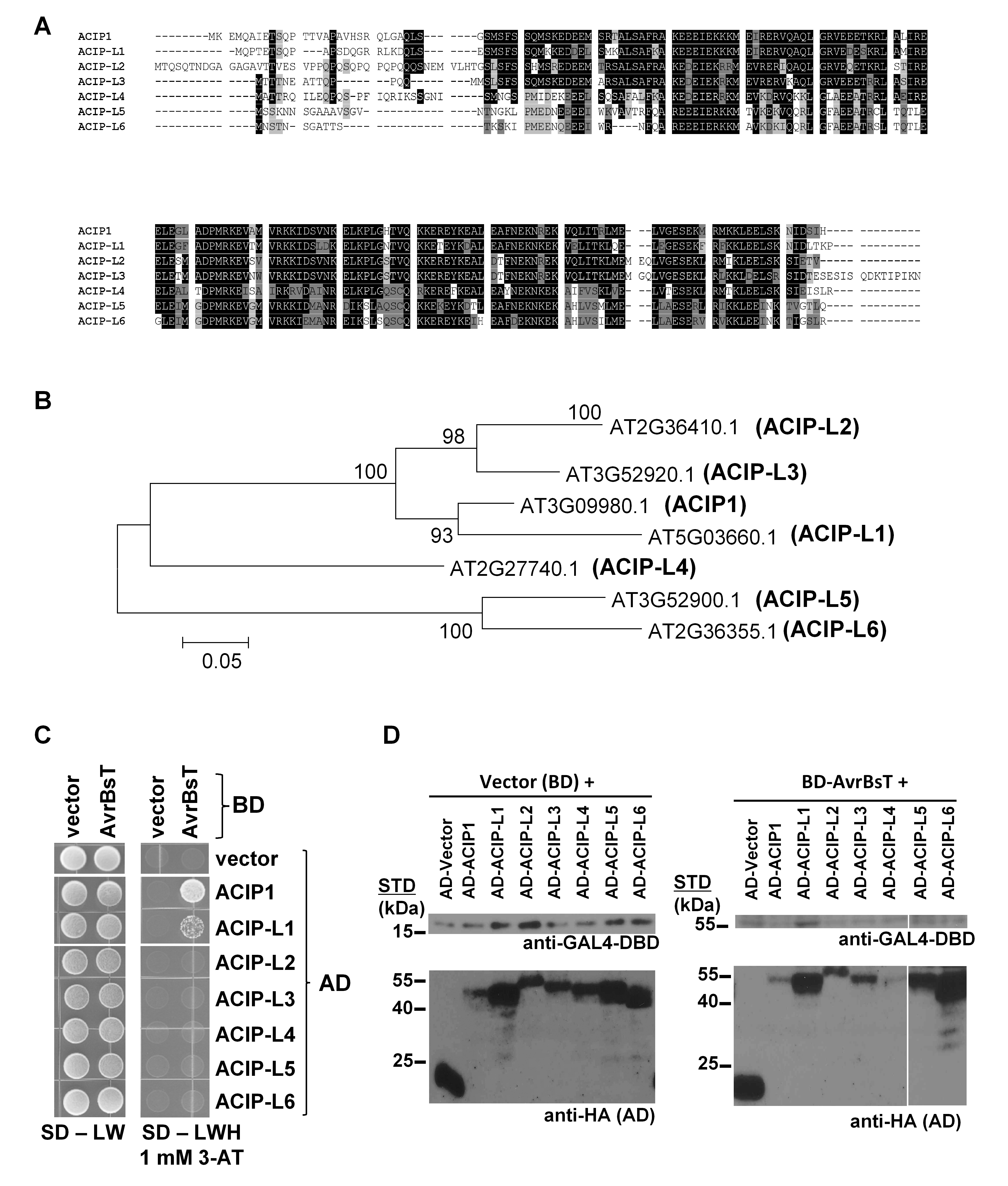

Supplement: Figure S1 — Arabidopsis ACIP protein family. (A) Amino acid sequence alignment of ACIP1 and six ACIP-like isoforms from Arabidopsis using Clustal W. (B) Tree of Arabidopsis ACIP protein family was generated by Neighbor-Joining method with default option (1000 bootstrap replicates and complete deletion for gaps/missing data) using MEGA4 software [61]. Bootstrap values are indicated in each branch and the bars represent branch lengths equivalent to 0.05 amino acid changes per residue. Arabidopsis gene numbers are listed next to assigned protein names. (C) Yeast two-hybrid assay showing AvrBsT binding to ACIP1 and ACIP-like isoforms. Yeast strains analyzed were AH109 carrying pXDGATcy86 (vector or AvrBsT) BD) and pGADT7(vector, ACIP1, ACIP-L1, ACIP-L2, ACIP-L3, ACIP-L4, ACIP-L5, or ACIP-L6). (D) Immunoblot analysis of proteins isolated from the yeast cells described in Figure 1A and Figure S1C. Analysis was performed using anti-HA sera to detect AD-HA or AD-HA-ACIP fusions and anti-GAL4DBD sera to detect BD or BD-AvrBsT. STD, molecular weight standard in kDa. Expected molecular weights: AD-HA-ACIP fusions = ∼38–41 kDa; BD = 16 kDa; BD-AvrBsT = 55 kDa. (TIF) [file ppat.1003952.s001.tif]

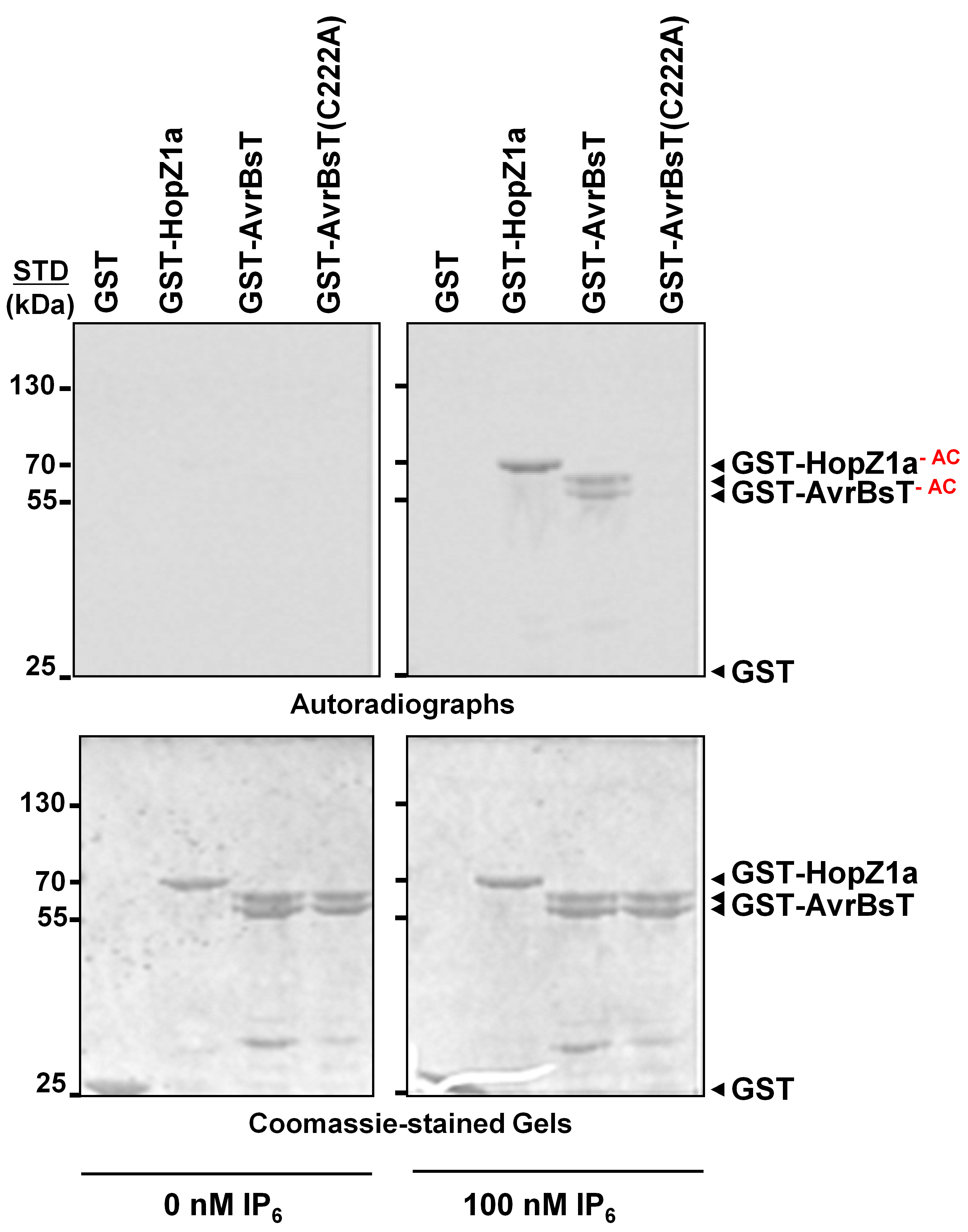

Supplement: Figure S2 — AvrBsT acetyltransferase activity is stimulated by inositol hexakisphosphate (IP6). Purified GST (negative control), GST-HOPZ1a (positive control), GST-AvrBsT, and GST-AvrBsT(C222A) proteins were incubated with 0.4 µCi 14C-acetyl CoA ±100 nM IP6 for 30 min at room temperature. Proteins were separated by 10% SDS-PAGE. Protein gel was stained with Coomassie and then analyzed by autoradiography. Acetylated proteins (GST-HopZ1a-AC and GST-AvrBsT-AC) are labeled in the autoradiograph. STD, molecular weight standard in kDa. GST = 28 kDa; GST-HopZ1a = 70 kDa; GST-AvrBsT = 65 kDa. (TIF) [file ppat.1003952.s002.tif]

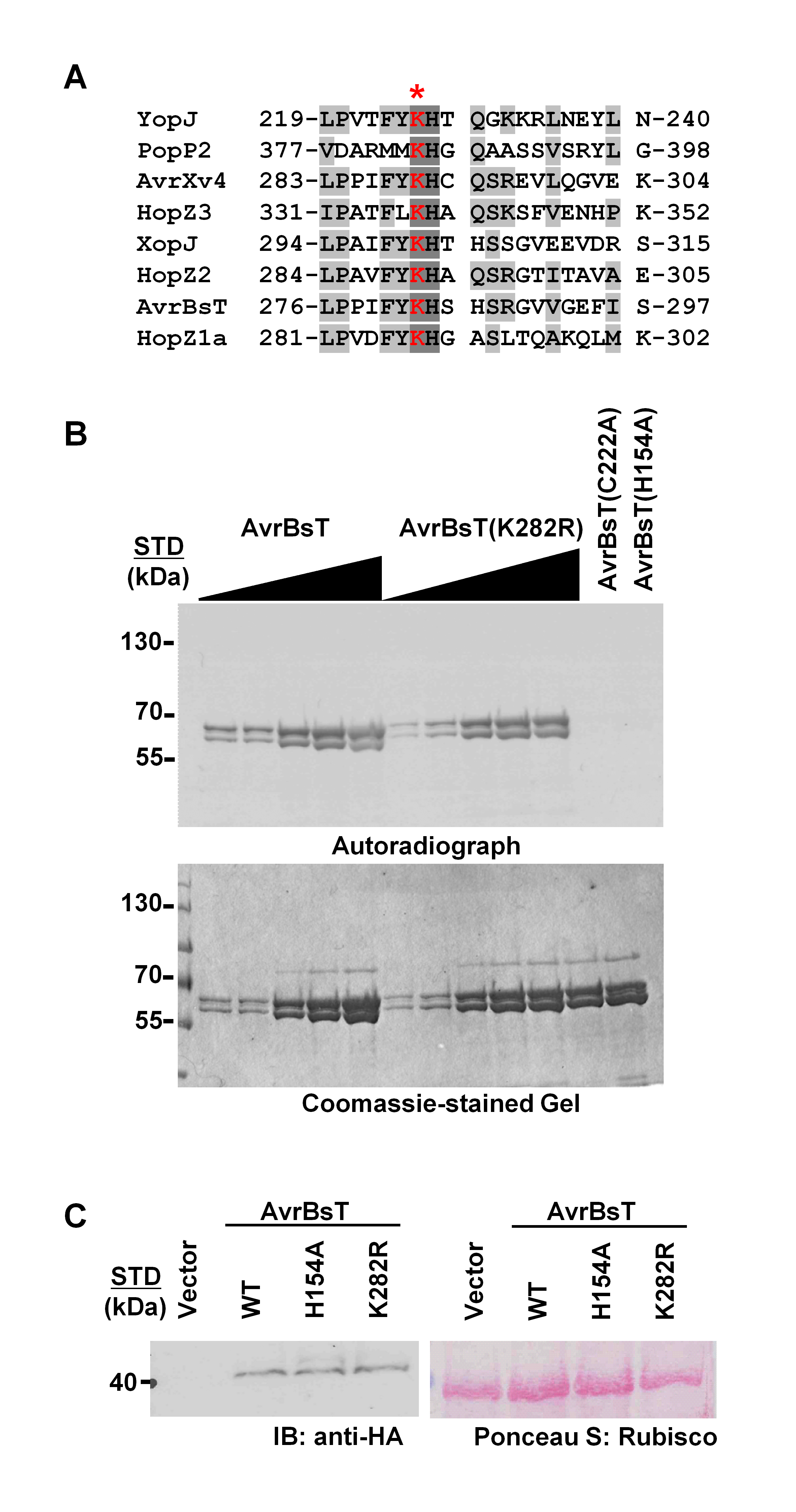

Supplement: Figure S3 — AvrBsT K282R mutant has auto-acetylation activity in vitro but exhibits reduced HR symptoms in planta . (A) Alignment of the conserved K282 residue (*) in AvrBsT with other homologs in the YopJ effector family: YopJ from Yersinia pestis, PopP2 from Ralstonia solanacearum, AvrXv4 and XopJ from Xanthomonas euvesicatoria, and HopZ1a, HopZ2, and HopZ3 from Pseudomonas syringae. (B) Auto-acetylation activity of AvrBsT(K282R) relative to wild type AvrBsT. Purified GST-AvrBsT(K282R) or GST-AvrBsT (0.5 to 5 µg) was incubated with 0.4 µCi 14C -Acetyl CoA and 100 nM IP6 for 1 hr at room temperature. GST-AvrBsT(C222A) and GST-AvrBsT(H154A) (5 µg) were used as negative controls. Proteins were separated by 10% SDS-PAGE. Gels were stained with Coomassie and then analyzed by autoradiography. STD, molecular weight standard in kDa. GST-AvrBsT = 65 kDa. (C) Wild type and mutant AvrBsT protein expression level in the Arabidopsis Pi-0 leaves described in Figure 2C. Proteins were detected by immunoblot analysis using HA sera. Ponceau S staining was used to detect Rubisco, which served as a loading control. (TIF) [file ppat.1003952.s003.tif]

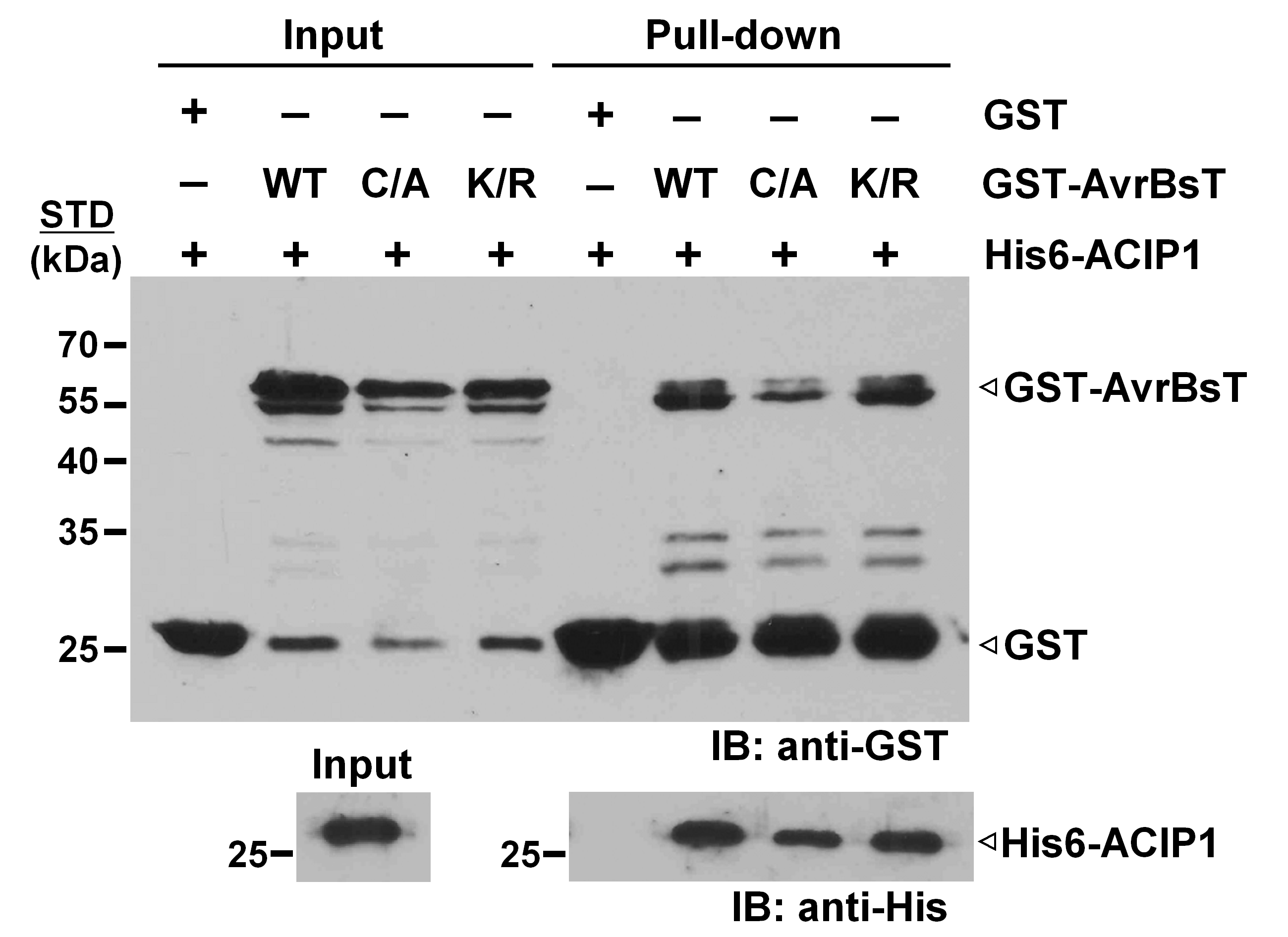

Supplement: Figure S4 — AvrBsT mutants interact with ACIP1 in vitro . GST-AvrBsT mutant protein affinity purification of His-ACIP1 in vitro. GST, GST-AvrBsT (WT), GST-AvrBsT(C222A) (C/A), or GST-AvrBsT(K282R) (K/R) was incubated with E. coli extracts containing His6-ACIP1. Proteins were purified by using glutathione sepharose and analyzed by immunoblot (IB) analysis using anti-GST and anti-His sera. Protein input is shown on left and pull-down on right. Expected protein MW = GST = 28 kDa; GST-AvrBsT mutants = 65 kDa; and His6-ACIP1 = 28 kDa. +, protein expressed,; −, vector control. STD, molecular weight standard. Similar phenotypes were observed in two independent experiments. (TIF) [file ppat.1003952.s004.tif]

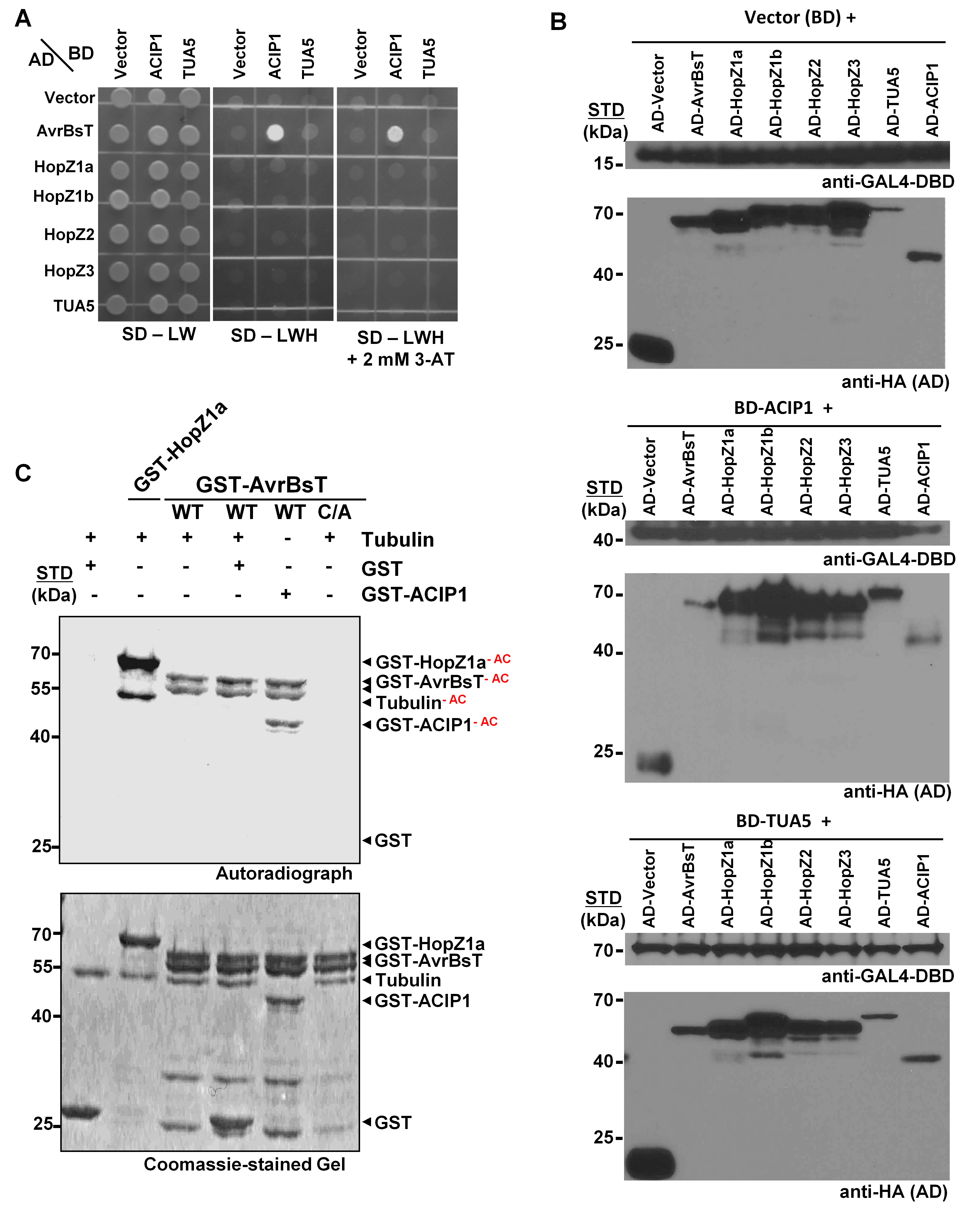

Supplement: Figure S5 — HopZ1a does not interact with or acetylate ACIP1 in vitro . (A) Candidate interaction test using yeast cells carrying AvrBsT, HopZ1a, HopZ1b, HopZ2, HopZ3, or TUA5 in pXDGATcy86(BD) and ACIP1 or TUA5 in pGADT7(AD). Strains were spotted on nonselective (SD –LW) and selective (SD –LWH ±2 mM 3-AT) media and then incubated at 30°C for 3 d. (B) Immunoblot analysis of proteins isolated from the yeast cells described in (A). Anti-HA and anti-GAL4DBD sera were used to detect proteins in pGADT7 and pXDGATcy86, respectively. Expected molecular weights: AD-vector = 23 kDa; AD-AvrBsT = 58 kDa; AD-HopZ1a = 63 kDa; AD-HopZ1b = 63.5 kDa; AD-HopZ2 = 60.5 kDa; AD-HopZ3 = 68.1 kDa; AD-TUA5 = 78 kDa; AD-ACIP1 = 43 kDa; BD = 16 kDa; BD-ACIP1 = 36 kDa; BD-TUA5 = 71 kDa. (C) HopZ1a acetylates tubulin but not ACIP1. Purified GST, GST-HopZ1a, GST-AvrBsT, or GST-AvrBsT(C222A) was incubated with Porcine tubulin (Cytoskeleton), GST, or GST-ACIP1 with 0.4 µCi 14C -Acetyl CoA and 100 nM IP6 for 1 hr at room temperature. Protein gels were stained with Coomassie and then analyzed by autoradiography. Acetylated proteins are labeled in the autoradiograph. GST = 28 kDa; GST-HopZ1a = 70 kDa; GST-AvrBsT = 65 kDa; tubulin = 55 kDa; GST-ACIP1 = 50 kDa. STD, molecular weight standard in kDa. (TIF) [file ppat.1003952.s005.tif]

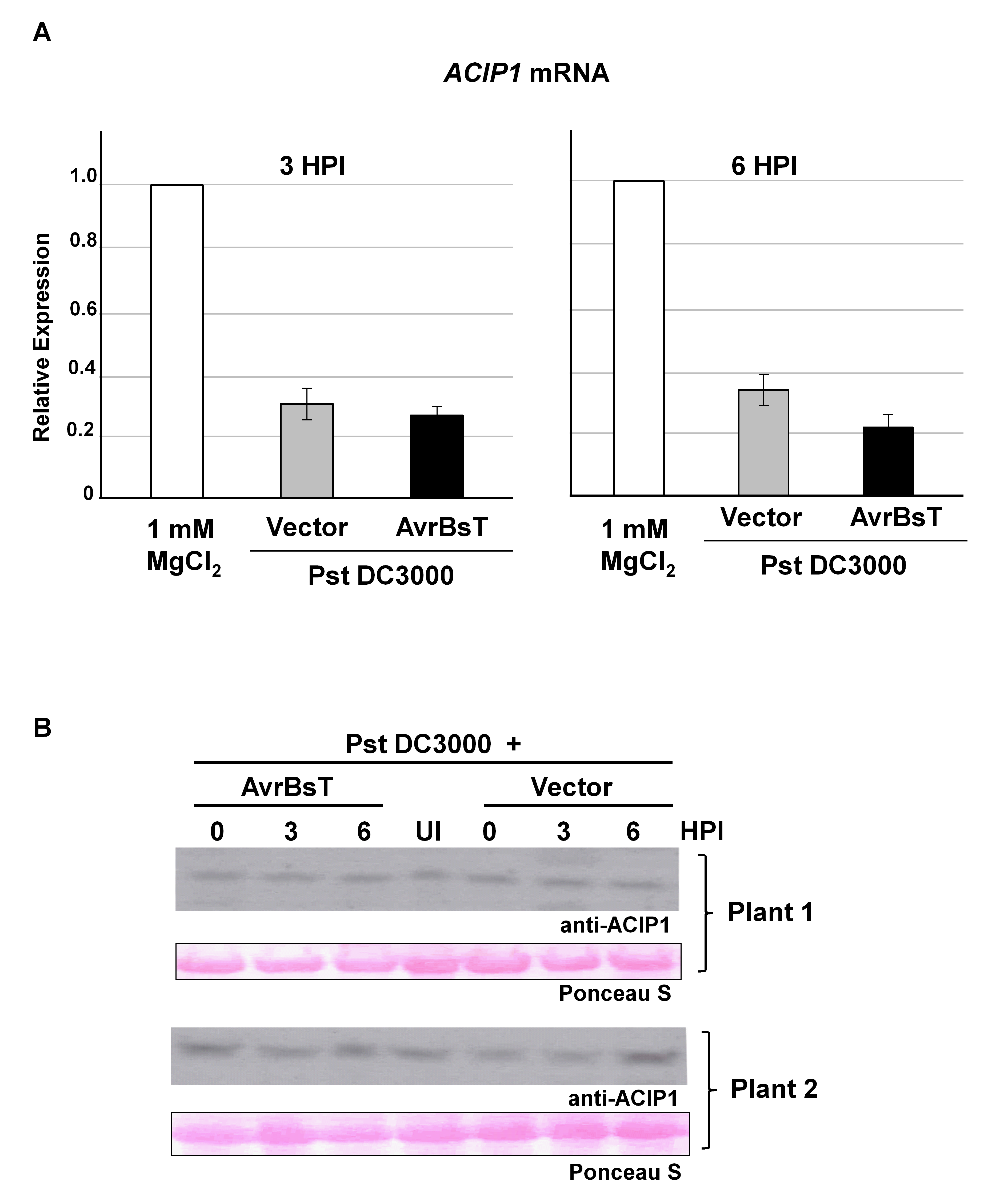

Supplement: Figure S6 — Levels of ACIP1 mRNA and protein abundance in Arabidopsis leaves during Pst DC3000 infection. Fully expanded Pi-0 leaves (n = 3 plants) were infiltrated with 1 mM MgCl2 (control) or a 2×10^8^ cells/mL suspension of Pst DC3000 alone (vector) or Pst DC3000 expressing AvrBsT (AvrBsT). (A) Relative expression of ACIP1 transcript at 3 and 6 HPI. Data were normalized using Actin8. Error bars represent ± SD. This experiment was repeated twice with similar results. (B) Immunoblot analysis of proteins isolated from two plants (1 and 2) inoculated with the strains described above. Anti-ACIP1 sera were used to detect endogenous protein expression. Ponceau S stained Rubisco large subunit was used as a loading control. (TIF) [file ppat.1003952.s006.tif]

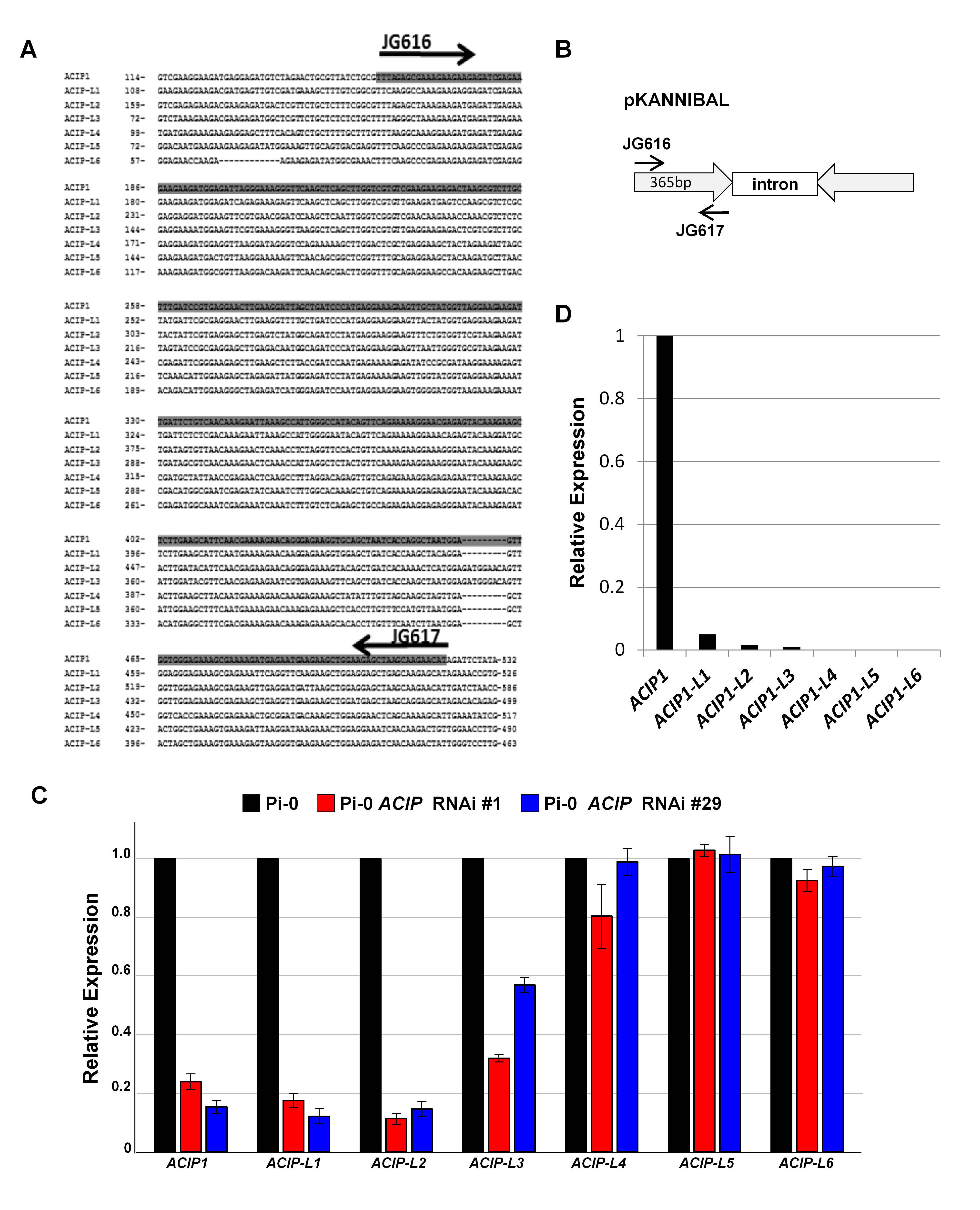

Supplement: Figure S7 — Transcript levels in Arabidopsis ACIP RNAi lines. (A) Alignment of the nucleotide sequence for ACIP gene family in Arabidopsis. Primers JG616 and JG617 were used to construct the hairpin ACIP RNAi construct in pKannibal (B) which was used to generate pART27(hp-ACIP). (C) ACIP1 and ACIP-like mRNA levels in Arabidopsis Pi-0 and Pi-0 ACIP RNAi lines #1 and #29. (D) Relative expression of ACIP1 and ACIP-like mRNAs in 4-week old, fully expanded leaves determined by qRT-PCR. For C and D, gene specific primers were used (Table S1). Data were normalized using UBQ5. Error bars represent ± SD. This experiment was repeated twice with similar results. (TIF) [file ppat.1003952.s007.tif]

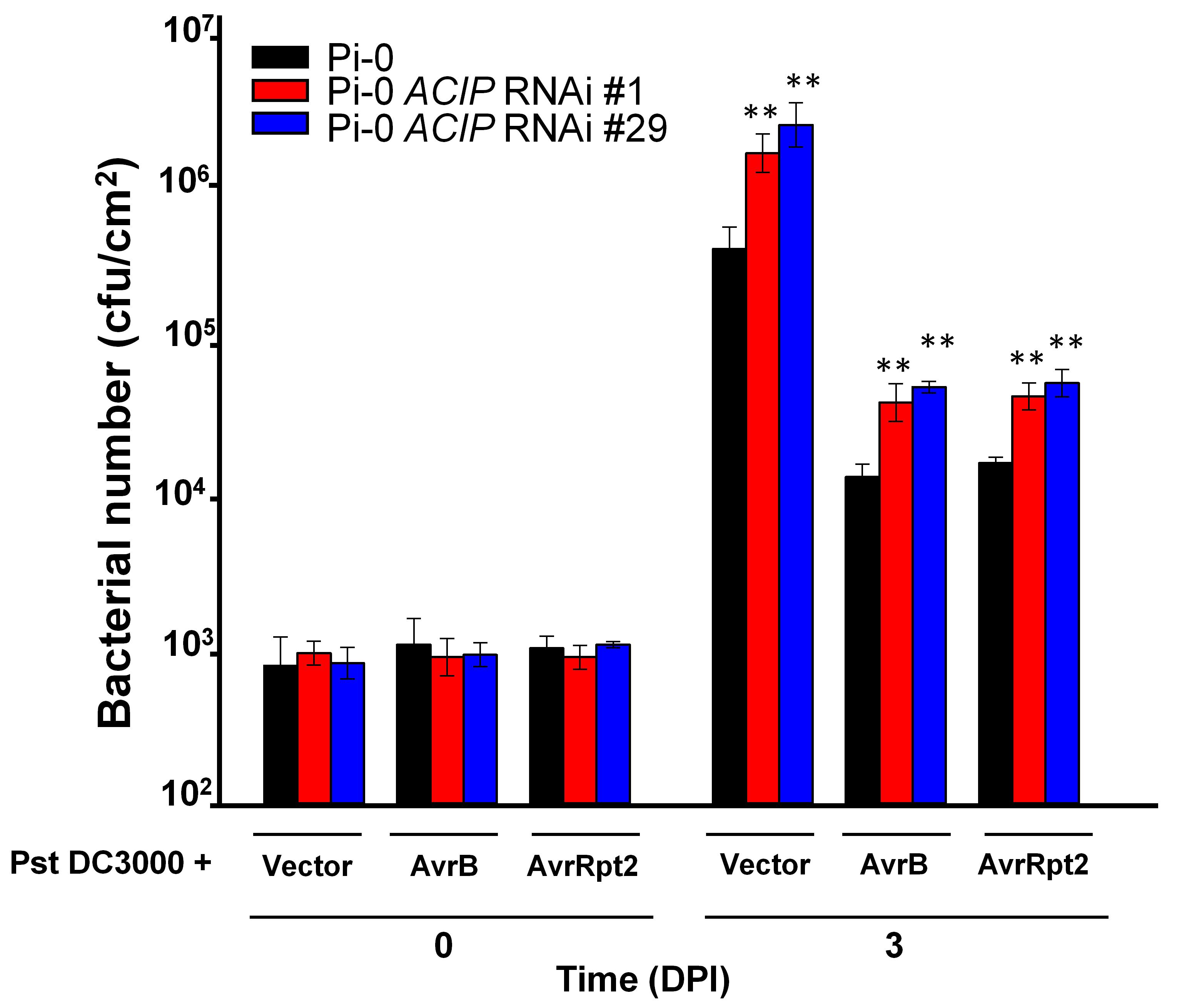

Supplement: Figure S8 — Members of Arabidopsis ACIP gene family are required for AvrB or AvrRpt2-triggered ETI. Increased growth of Pst DC3000, Pst DC3000 AvrB, or Pst DC3000 AvrRpt2 in Pi-0 ACIP RNAi line #1 (red bars) and line #29 (blue bars) compared to wild-type Pi-0 (black bars). Leaves were syringe-infiltrated with a 1×10^5^ cells/mL suspension of bacteria. Titers were assessed at 0 and 3 days post-inoculation (DPI). Data are mean cfu/cm2 ± SD (n = 4). Asterisks indicate statistically significant differences from Pi-0 (student t-test, **p<0.01). Experiment was repeated twice with similar results. (TIF) [file ppat.1003952.s008.tif]

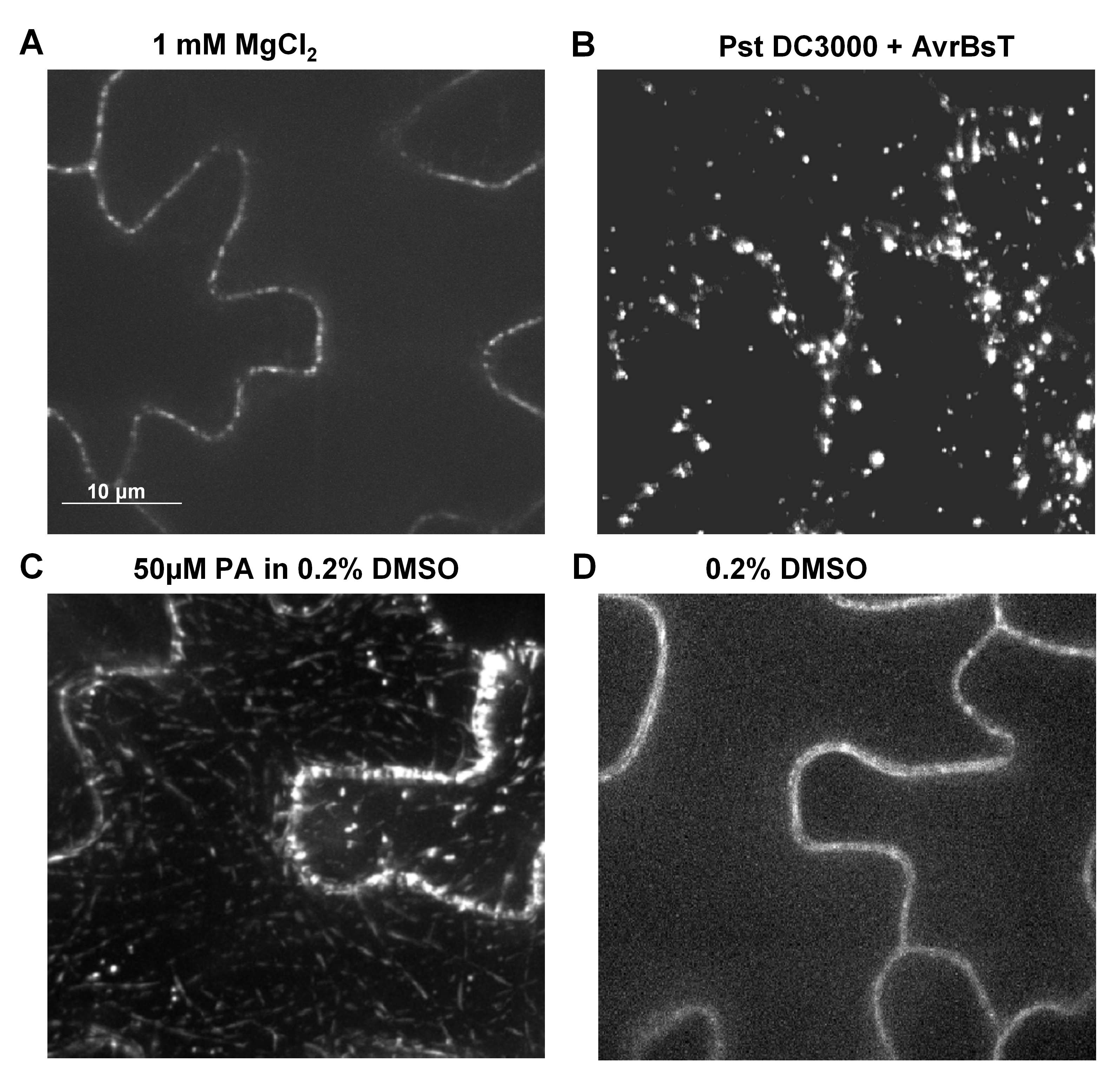

Supplement: Figure S9 — GFP-ACIP1 localization changes in response to different treatments. Pi-0 PACIP1::GFP-ACIP1 leaves were inoculated with: (A) 1 mM MgCl2, or a 3×10^8^ cells/mL suspension of (B) Pst DC3000 AvrBsT, (C) 50 µM PA in 0.2% DMSO or (D) 0.2% DMSO. Spinning disk confocal images were recorded at 6–7 HPI (A–B) or 1.5 HPI (C–D). Bar = 10 µm. Similar results were obtained in more than 3 independent experiments. (TIF) [file ppat.1003952.s009.tif]
