# Supplementary material for: AvrBsT Acetylates Arabidopsis ACIP1, a Protein that Associates with Microtubules and Is Required for Immunity
Source: PLoS Pathog. 2014 Feb 20;10(2):e1003952. doi: 10.1371/journal.ppat.1003952 (PMC3930583; doi:10.1371/journal.ppat.1003952)
Supplement: Table S1 — Primer sequences used in this study, related to Experimental Procedures. (DOCX) [file ppat.1003952.s010.docx]

**Table S1. Primer sequences used in this study, related to Experimental Procedures.**

| **Description** | **Primer Name; Sequence (5'→3')** |
| --- | --- |
| **for cloning cDNAs and genomic sequences**  *avrBsT*  *ACIP1*  *ACIP1-LI*  *ACIP1-L2*  *ACIP1-L3*  *ACIP1-L4*  *ACIP1-L5*  *ACIP1-L6*  *ACIP1 promoter*  *hopZ1a*  *hopZ1b*  *hopZ2*  *hopZ3*  *TUA5* | KP 55;ATGAAGAATTTTATGCGTTCACTTGGC  SC 28;TGATTCAATAGTTTTCCTAATT  KP 40; ATGAAGGAGATGCAGGCAATAGAGACG  KP 42; GTGTATAGAATCTATGTTCTTGCTTAGCT  AG 09; ATGCAACCGACAGAGACG  AG 10; AGGTTTGGTTAGATCAATGTT  AG 01; ATGACTCAGAGTCAAACCA  AG 02; CACGGTTTCTATGCTCTT  AG 03; ATGACAACAACGAATGAAGC  AG 04; GTTCTTGATTGGTATGGTCTT  JG588; ATGGCTACGACAAGACAGATCTTG  JG589; GCGTAGCGATATTTCAATGCTTTT  JG590; ATGAGTAGCAAGAATAACTCCGG  JG591; TTGCAAGGTTCCAACAGTCTTGT  AG 08; ATGAACAGTACCAACTCAGG  AG 06; TCGCAAGGACCCAATAGTC  AK128; CTCAGTTTGATCACAAATAAA  AK129; AGAAATAGTGCTCTGATA  JG626; ATGGGAAATGTATGCGTCGG  JG627; GCGCTGCTCTTCGGCAAGTAC  JG628; ATGGGAAATATATGCATCGGCGG  JG629; GCCCTGAGCCGCAGCCAATA  JG630; ATGGGAATTTGCGTTTCCAAACC  JG631; TTGGCTCTCTAGGGCTTTCCTTAT  JG632; ATGAATATCTCAGGTCCGAACAGA  JG633; AGGCTTGGCCCGGACCCTGTTT  KP 51; ATGAGGGAAATTATTAGCATTCATAT  KP 52; ATAGTCTTCACCTTCATCTTCTTC |
| **for GST fusion proteins**  GST-AvrBsT | MB225; GGGATCCCCATGAAGAATTTTATGCGTTC  MB185; CTCGAGTTATGATTCAATAG |
| **for ACIP1 RNAi construct** | JG616; CCTCGAGTTTAGAGCGAAAGAAGAAGAGATC  JG617; GGAATTCATGTTCTTGCTTAGCTCTTCCA |
| **for site-directed mutagenesis**  AvrBsT(K282R) | KP 73; GCCGCCCATTTTTTATAGGCATTCACACTCTAGAGGGG  KP 74; CCCCTCTAGAGTGTGAATGCCTATAAAAAATGGGCGGC |
| **Quantitative RT-PCR**  *ACIP1*  *ACIP1-LI*  *ACIP1-L2*  *ACIP1-L3*  *ACIP1-L4* | JG606; CTATACACTGACTTTGAATCCAAGACAG  JG607; CCCCTAACAAAGATAATAATTGTAGTAGTAA  JG608; GTGTTGGACGAGCAGAGTCG  JG609; CTTTGCAACATCTAGTTCAAACACATA  JG610; GTGTGAAAAAGGTTGTTCAAGAAGA  JG611; CCACAAATTTTACAAATCAAACCC  JG810; AGAGTGAATCTATCTCACAAGACAAGAC  JG811; ACACCCAGAAACTAAGAAAACATTC  JG812; TGAAATATCGCTACGCTAACACA  JG813; CTACTAAGGTTACTGAATCAGGAAAACA |
| *ACIP1-L5*  *ACIP1-L6*  *UBQ5*  *ACTIN8*  *WRKY22*  *WRKY29* | JG814; GGAACCTTGCAATGAGGAGAAGC  JG815; TATCGTACATCCTGTTACATCGTAGC  JG816; CTATTGGGTCCTTGCGATAATC  JG817; GGTATGAATTTCTTAAATCCCTGAGG  MS82; GTGGTGCTAAGAAGAGGAAGA  MS83; TCAAGCTTCAACTCCTTCTTT  JG612; CCCAAAAGCCAACAGAGAGA  JG613; CATCACCAGAGTCCAACACAAT  MS108; TCCTTCGGAGAGATTCGAGA  MS109; CTGCTGCTACATGGCACACT  MS 88; GCGTAACGGGCAGAAAC  MS 89; GGTTTGGGTTGGGAAGTTTT |
|  | |
